# Assessment of ICount software, a precise and fast egg counting tool for the mosquito vector *Aedes aegypti*

**DOI:** 10.1186/s13071-016-1870-1

**Published:** 2016-11-18

**Authors:** Julie Gaburro, Jean-Bernard Duchemin, Prasad N. Paradkar, Saeid Nahavandi, Asim Bhatti

**Affiliations:** 1 Institute for Intelligent Systems Research and Innovation (IISRI), Deakin University, 75 Pigdons Road, Waurn Ponds, VIC 3216 Australia; 2 CSIRO Health & Biosecurity, Australian Animal Health Laboratory, 5 Portarlington Rd, East Geelong, VIC 3220 Australia

**Keywords:** *Aedes aegypti*, ICount, Automatic/semi-automatic egg counting, P-cresol

## Abstract

**Background:**

Widespread in the tropics, the mosquito *Aedes aegypti* is an important vector of many viruses, posing a significant threat to human health. Vector monitoring often requires fecundity estimation by counting eggs laid by female mosquitoes. Traditionally, manual data analyses have been used but this requires a lot of effort and is the methods are prone to errors. An easy tool to assess the number of eggs laid would facilitate experimentation and vector control operations.

**Results:**

This study introduces a built-in software called ICount allowing automatic egg counting of the mosquito vector, *Aedes aegypti*. ICount egg estimation compared to manual counting is statistically equivalent, making the software effective for automatic and semi-automatic data analysis. This technique also allows rapid analysis compared to manual methods. Finally, the software has been used to assess p-cresol oviposition choices under laboratory conditions in order to test the system with different egg densities.

**Conclusions:**

ICount is a powerful tool for fast and precise egg count analysis, freeing experimenters from manual data processing. Software access is free and its user-friendly interface allows easy use by non-experts. Its efficiency has been tested in our laboratory with oviposition dual choices of *Aedes aegypti* females. The next step will be the development of a mobile application, based on the ICount platform, for vector monitoring surveys in the field.

**Electronic supplementary material:**

The online version of this article (doi:10.1186/s13071-016-1870-1) contains supplementary material, which is available to authorized users.

## Background

Vector-transmitted pathogens, especially those transmitted by mosquitoes, are a major burden on human health. The mosquito *Aedes aegypti* is a potential vector of viruses such as dengue, chikungunya, and yellow fever viruses. A recent study estimated that this vector is responsible for hundreds of millions of human infections and over 50,000 human deaths annually [1]. *Aedes aegypti* is a threat for potential emerging diseases and is considered to be one of the main vectors of the recent Zika virus outbreak in Brazil, associated with a high number of microcephaly cases in infants [2].

Vector monitoring is one of the main strategies to assess the impact of vector control operations and vector-borne agent dissemination. *Aedes aegypti* is rarely found far from human habitation and can oviposit in a wide range of man-made containers [3]. The eggs attach to solid substrates, close to the water’s edge, and generally hatch when submerged. One of the major problems concerning arbovirus transmission by *Aedes* mosquitoes is that eggs can survive several months under dry conditions without desiccation [3]. In order to regulate the number of eggs laid and limit vector’s transmission rate, it is important to understand and foresee which sites females prefer to lay their eggs. One of the aims of vector control strategies is to detect larval sites as fast as possible to guide environmental treatments to reduce vector populations. For this purpose, it is important to scan as many geographical sites as possible in high arbovirus circulation zones.

The ecology and behaviour of vectors, which determine population abundance and transmission risk, are key factors for controlling virus dissemination [4]. Fecundity is often used as a marker to assess population fitness of mosquitoes, and this has been validated in field studies [5, 6]. Female mosquito’s fecundity, one of the most important traits to estimate their fitness, can be determined by counting the number of eggs laid [7].

*Aedes aegypti* mosquitoes lay approximately between 20 and 140 eggs per blood meal depending on the amount of blood taken, body size reserves and fecundity of the female [8]. The eggs are laid one by one on a solid support [9] allowing simple egg sampling. However, quantifying eggs routinely is time consuming and, more importantly, prone to errors if done manually by eye counting. Experiments in the laboratory and monitoring surveys in the field could involve hundreds of samples. New tools for automatic data acquisition, such as egg counting, would improve the speed and accuracy of these operations.

Recent technological advances allow processing of digital data to automatically extract important behavioural information to understand ecological systems [10]. Those new techniques now enable us to collect large and accurate data sets, and also detect behaviours previously undetectable by the human eye when observing high numbers of individuals. For example, mosquito flight behaviours are not completely understood and the development of algorithms are crucial to access this new types of data (trajectory, overall activity) [11]. Methods have recently been developed for automatized counting of *Ae. aegypti* eggs via digital image analysis [12–16]. However, some of these techniques require prior knowledge of the algorithm and the image processing techniques employed [13, 15]. Other techniques require cumbersome hardware including scanners, cameras, LED lighting systems and mechanical support equipped with a motorized linear translation stage [14, 16].

In this paper, we introduce simple software (ICount) which has the capacity to count *Ae. aegypti* eggs using automatic and semi-automatic methods. The semi-automatic method is defined by Barbedo [17] as an automated process with a human input to refine the estimates. The main advantage of ICount is its user friendly interface and free access to download [18] (free test images are also available at this link).

In order to test ICount software’s operability in a laboratory context, oviposition choice experiments have been carried out in our lab. To choose their oviposition site, females use olfactory cues, in addition to visual and tactile information [3]. Some semiochemicals have “oviposition attractant” properties making females orientate their flight in their direction. Other semiochemicals can have an “oviposition repellent” effect causing females to fly away from the substrate [19]. We used the semiochemical 4-methylphenol or p-cresol, at various concentrations, known to either attract or repulse gravid female *Ae. aegypti* to lay eggs depending on the chemical abundance. P-cresol is a volatile compound isolated from the extract of fermented Bermuda grass (*Cynodon dactylon*) successfully identified by Millar et al. [20] and is associated with *Culex* mosquito oviposition. The effect of p-cresol on *Ae. aegypti* oviposition choice has been tested but remains ambiguous. Indeed, previous research has shown that Bermuda infusions either repel [21] *Ae. aegypti* females or attract them [9]. P-cresol has shown repellent effects at 10^-5^ and 10^-3^ ppm (or concentrations of 0.01 and 1.0 μg/l, respectively) [22] but also attractive effects at 4.10^-5^ ppm (or 0.04 μg/l) [23]. No effects were found at 10^-1^ ppm (or 100 μg/l).

We also investigate the effects of p-cresol on oviposition choices at different concentrations to resolve ambiguous data from the literature. By assessing the eggs laid, we tested the traditional manual counting method against our developed software to determine the precision (sensitivity) of ICount. In the methods, we provide a short introduction and indications of the use of the software.

## Methods

### Mosquito colony maintenance

*Aedes aegypti* mosquitoes from Cairns, Queensland (about 8th generation) were reared in Plexiglas cages (30 × 30 × 30 cm) within a colony room maintained at 26 °C and 65% relative humidity. The room was set at a daily photoperiod of 12:12 (L:D). Insects were fed on sugar ad libitum and females aged from 2 to 5 weeks old received a weekly blood meal.

### Membrane feeding and egg sampling

Chicken blood meal was offered via chicken skin membrane with an artificial blood feeder (Hemotek®, Accrington, UK) once a week for 1 h. Three days after the blood meal, glass beaker containers were placed inside the cage for females to lay eggs. A sandpaper strip, the length of a 100 ml beaker’s perimeter (160 mm), was put inside the container which was filled with water (50 ml) up to half the strip width (25 mm). Once females finished laying the eggs, up to 5 days after the blood meal, the strip was removed from the colony cage for air drying at room temperature.

The same setup was used for semiochemical assays. Gravid females were given a dual choice between water (control) and p-cresol at different concentrations in aqueous solution for egg laying. The beakers are placed at opposite corners of the cage and rotated every 24 h. Four concentrations of p-cresol were tested (10^-1^ ppm, 10^-2^ ppm, 10^-4^ ppm and 10^1^ ppm); p-cresol at 10^-1^ppm, as control, should not trigger any choice difference with water alone for *Ae. aegypti* females [22]; p-cresol at 10^-2^ ppm and 10^1^ ppm as this point is missing in the literature; p-cresol at 10^-4^ppm as published results are ambiguous [22, 23].

### Egg image processing

Images of the sandpaper strip with eggs can be taken any time after collection: directly after drying or it can be stored in a dry place until data sampling. Two types of image were sampled. An overall image of the paper was taken using a camera (Canon, EOS6D) with the focus in the plane of the eggs. This set of images will be referred further as “macro”. The second type of image was taken employing a Leica microscope (DFC425) using 8.0× magnification with a 1.0× objective, and referred as “micro”. An average of 8 “micro” images was needed to cover the whole sandpaper strip egg area. The images taken with the camera were trimmed with Paint software (Microsoft Windows) in order to remove the background. The pictures taken with the microscope camera did not require any processing and could be directly processed in ICount software.

### Manual counting

As a gold standard reference method to confirm the accuracy of ICount software, the eggs were counted manually by using ImageJ software and the “Cell Counter” plugin [24] which allows automatic summation of manually marked objects on the image. The “micro” pictures were used for the manual counting method. By determining the sum of manually counted eggs, the total number of eggs laid after each blood meal could be estimated for each corresponding full sand strip.

### Egg estimation using ICount software

**Fig. 1:**
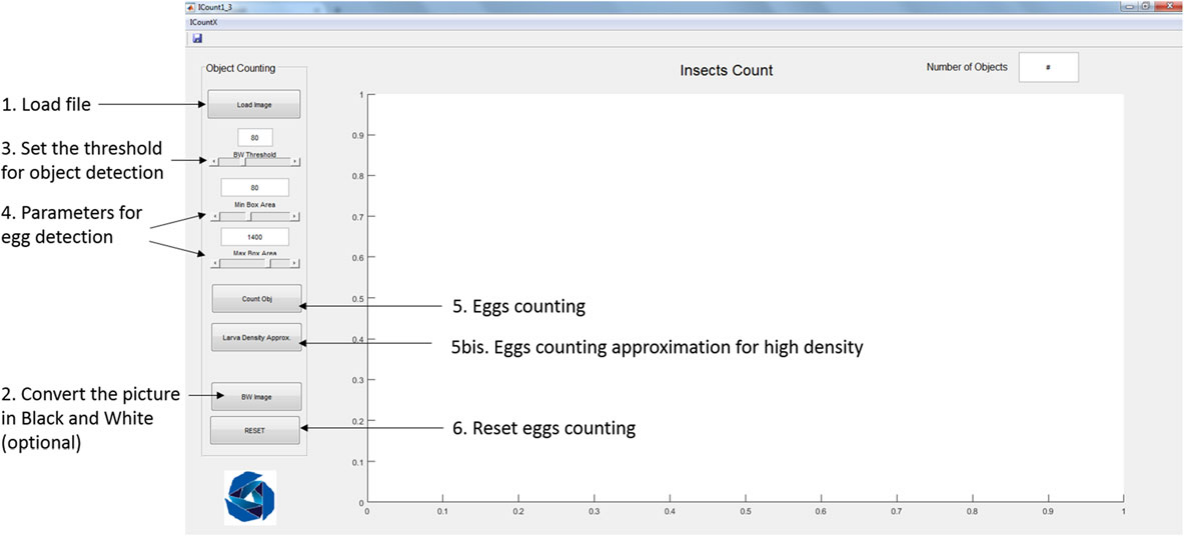
ICount interface before loading the file

For automatic counting, “macro” and “micro” images were analysed using ICount. The egg counting steps were visualised on the software shown interface in Fig. 1 with the following options selected: uploading the image (step 1) setting the parameters for object detection (steps 3 and 4) and starting the count. There are three input parameters defined in the software, i.e. black and white (BW) Threshold, minimum (Min) and maximum (Max) Box area. BW Threshold defines the binary cut-off value to convert a grayscale image into black and white and takes the value within the range of [0 255], where 0 represents black and 255 represents white. Box area parameters define the approximate area covered by a single egg in pixels. Default values of 80, 800 and 1400 for BW Threshold, Min and Max Box Area, respectively were used if not tuned by the user.

**Fig. 2:**
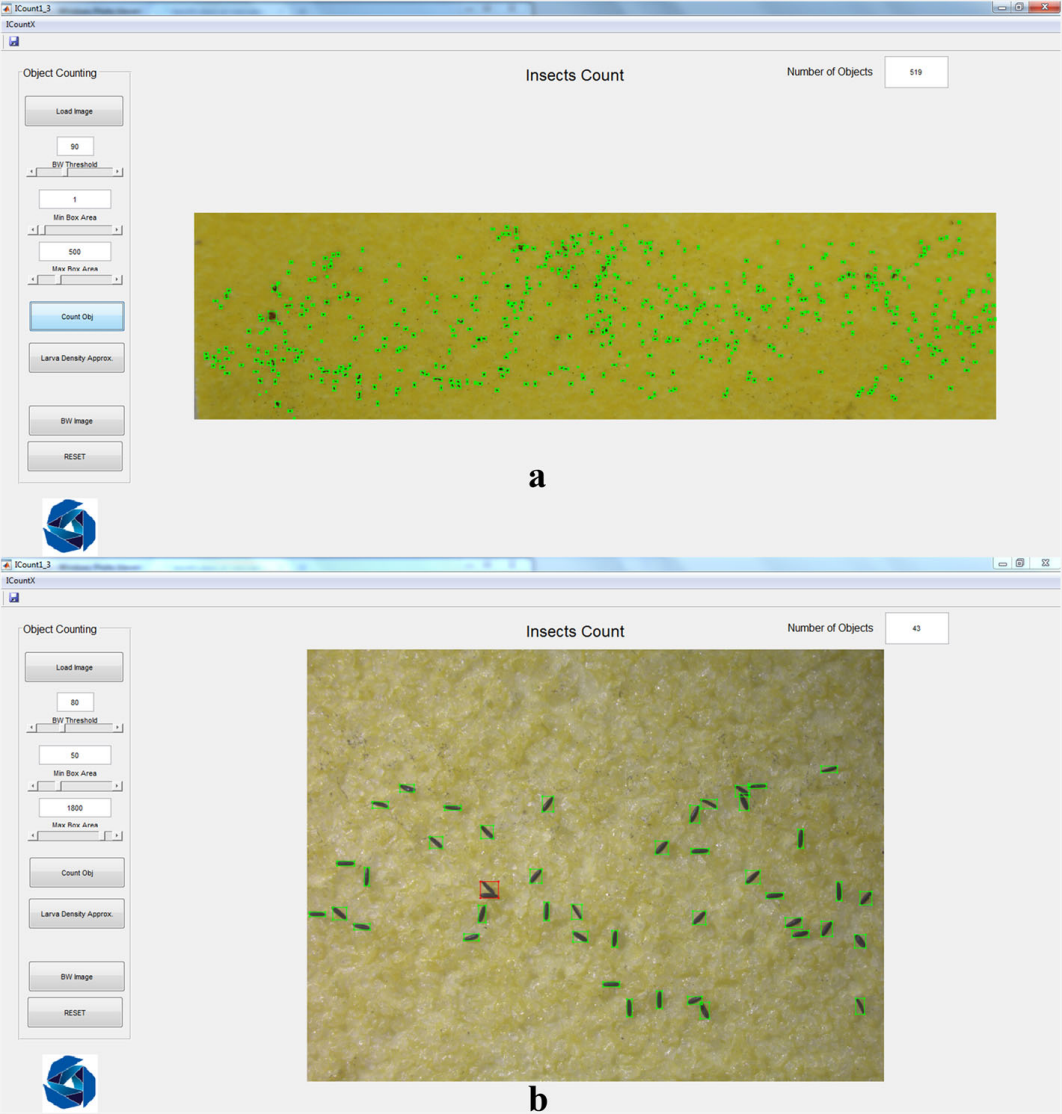
ICount interface with two types of image. **a** “Macro” image showing the overall sandpaper strip for “macro” analysis; it provides an estimation of the number of eggs laid employing colour histogram. **b** “Micro” image for “micro” analysis, providing a precise number of eggs laid via the snapshots taken under the microscope

For semi-automatic egg counting, the user can check the estimation made by the computer by checking the eggs counted automatically. As shown in Fig. 2, red and green boxes appear after the estimation. Green boxes indicate that the object is perfectly detected by the algorithm, and red boxes indicate possible errors or uncertainty of the software for the object. This can happen when the density of eggs laid on the sand strips is too high: more than 100 eggs per “micro” image or more than 1000 eggs per “macro” image. Another software error can arise from eggs laid too close to each over, then overlapping and counted as a single object by the algorithm (see Additional file 1: Figures S1 and S2). The user can then correct this uncertainty by modifying the total objects counted while the pictures are processed. This method allows correction of the error estimation and accurate counting.

Oviposition sandpaper strips of different size and different numbers of eggs from different blood meals were analysed. Manual counting via ImageJ and automatic counting via ICount were compared by generating calibration curves for both methods.

In order to evaluate the time efficiency of our software, we measured the time needed to analyse a whole sand strip for the automatic, semi-automatic and manual methods with three different egg densities from high (80–200 eggs), medium (30–80 eggs), and low (10–30 eggs). We also measured the time required when using a previous study method that employs ImageJ plugin to automatically count *Ae. aegypti* eggs (for more details, see methods of Afify & Galizia [22]). Briefly, the image was converted into an 8-bit format and a manual threshold was applied to the image in order to differentiate eggs from the background. The egg number was then estimated with the “Particle counts” plugin. As explained in their study, the number of eggs on the filter paper was calculated as the ratio of the two readings of total area and average area of the individual eggs (“Analyse particles” function run twice).

For ‘micro’ images, the results from analyses in two different experimenters were averaged for each of these different egg-counting methods. Timing commenced when clicking on the “load” button of ICount or ImageJ and concluded when the total egg number was counted or estimated by the software.

### Statistical analysis

Manual and automatic counting data were compared using R for statistical analysis [25] by employing the lm function. The calibration curve is generated by plotting together “Manually egg count” (reference) and “Estimated egg count” (results from ICount) data and tested against the linear model Y = a + bx. A total of 30 blood meals was given to different cages over several months. In total, we obtained 48 “macro” images from the whole strips. Each sand strip required an average of 8 images with the microscope to cover the whole area where the eggs were laid. In that case, a mean of 380 (48 × 8) pictures was taken for the “micro” analysis.

For p-cresol assays, each different concentration of p-cresol was analysed in triplicate over 3 different colony cages randomly interchanged between the different assays. The “micro” images were used for data analysis using the semi-automatic method. In total, about 16 images were sampled for each p-cresol assay. Egg number data are transformed in percentage for p-cresol dual choice comparisons with the formula: $Egg(choice)\%=\frac{\mathrm{eggs}\left(\mathrm{choice}\right)\#}{\mathrm{total}\ \mathrm{eggs}\ \#}\ast 100$. Data distribution for each p-cresol assay was normal (Shapiro test *P* > 0.05), so parametric “Welch Two Sample *t*-tests” were used to define the attractive or repulsive effect of p-cresol concentrations. Finally, a three-way ANOVA model was built with the factors “Choice”, “Date” and “Egg number” using the packages “car”, “effects” and “multicomp”. The “Choice” parameter represented the dual choice of females between p-cresol and water. The “Egg number” parameter is the number of eggs laid in total on each sand strip to see if the egg number influences female’s choice. The influence of the date, and so the different blood meal, was tested using the “Date” parameter.

## Results

### ICount analysis

**Fig. 3:**
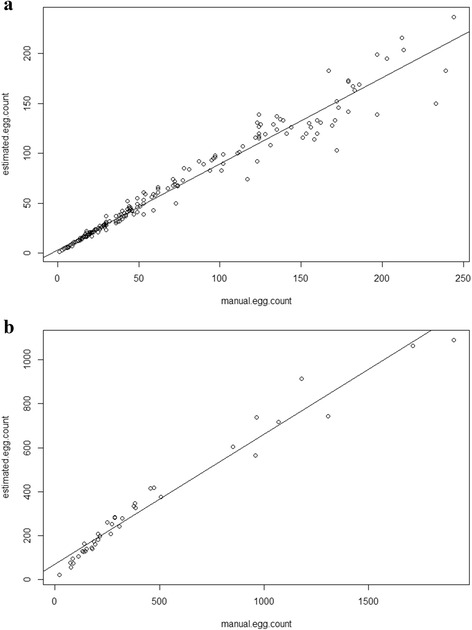
Validation of ICount egg counting approach. **a** Calibration curve of Manual *vs* Automatic counting of microscopic pictures of eggs (*n* = 380). Y_micro_ = 0.87x_micro_ + 3.02; *R* ^2^ = 0.965, *P* < 0.0001. **b** Calibration curve of Manual *vs* Automatic counting of camera “macro” images of eggs (*n* = 48). Y_macro_ = 0.59x_macro_ + 69.74, *R* ^2^ = 0.965, *P* < 0.0001

After generating calibration curves using both methods, we found that the *P*-values of the regression lines were highly significant (Y_micro_ = 0.87x_micro_ + 3.02; *R* ^2^ = 0.965, *P* < 0.0001 and Y_macro_ = 0.59x_macro_ + 69.74, *R* ^2^ = 0.965, *P* < 0.0001). This indicated that the manual versus automatic counting methods showed no statistically significant difference with the two types of image (Fig. 3).

The “Micro” analysis had a statistically higher value for the egg density for less than 100 eggs per image (Y_micro (eggs≤100)_ = 0.96x_micro (eggs≤100)_ + 0.22; *R* ^2^ = 0.979, *P* < 0.0001); however, the automatic method was less strong with more than 100 eggs on the same image (Y_micro (eggs>100)_ = 0.79x_micro (eggs>100)_ + 13.84; *R* ^2^ = 0.686, *P* < 0.0001, see Fig. 3a). This is due to egg superimposition occurring more often when the density is high.

On average, the manual method was about 19 times slower than the automatic method and about 10 times faster than the semi-automated method for high density of eggs (Table 1). For the medium density, the manual method takes 13 and 8 times longer than manual and semi-automatic methods, respectively. Finally, for low density eggs, the manual and semi- automatic methods are about 3 times faster than the manual method. In practice, about 5 min are required to count a whole sand strip (of average 8 “micro” pictures) compared to half a minute when using the automatic method. ICount is also more time-efficient than the other automatic counting method used with ImageJ, which needed on average 7 times more time than the automatic method using our software (column “ImageJ” in Table 1). This is due to the image conversion and the threshold adjusting on each individual picture before automatic counting.

**Table 1:** Time estimation (mean ± standard deviation in seconds) for image analysis with different methods and software: from ICount (automatic and semi-automatic) and ImageJ (manual and automatic)

	Automatic (ICount)	Semi-automatic (ICount)	Manual (ImageJ)	Automatic (ImageJ)
High density	3.8 ± 1.4	7.2 ± 3.0	69.5 ± 20.1	20.04 ± 0.53
Middle density	3.8 ± 1.9	6.0 ± 1.6	49.3 ± 14.8	17.26 ± 0.3
Low density	4.7 ± 2.7	3.7 ± 1.3	11.8 ± 7.1	16.14 ± 1.18

### P-cresol preference assay

**Fig. 4:**
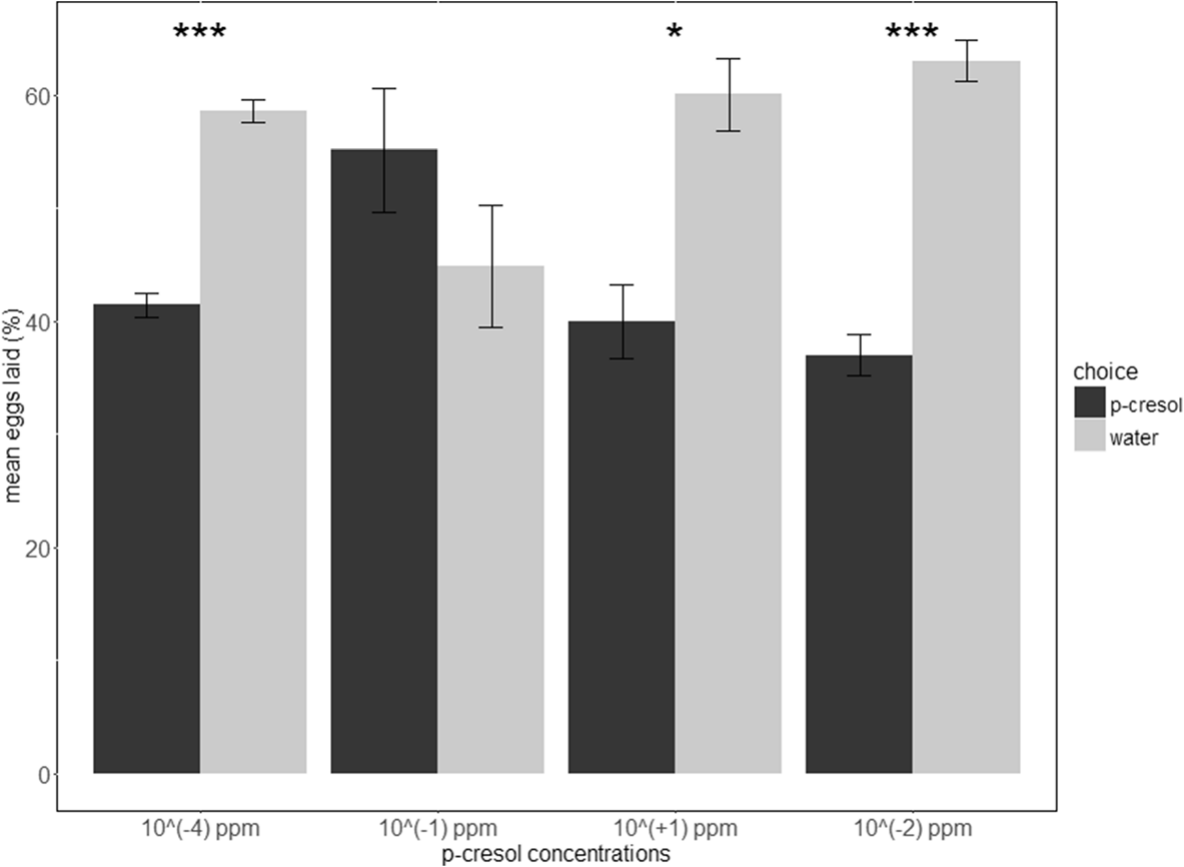
Bar plot of female mosquito choices at different concentrations of p-cresol assays using the semi-automatic method of analysis for egg counting (*n* = 24). *P = 0.0118, ***P_(10^ (-4) ppm)_ = 0.0003 and ***P_(10^ (-2) ppm)_ < 0.0001

The effect on oviposition of different concentrations of p-cresol against water was tested with *Ae. aegypti* female mosquitoes. For the analysis, we used the semi-automatic method of ICount with “micro” images. Figure 4 illustrates the results of female preferences during the assays. At 10^-1^ ppm, there was no preference between p-cresol and water for females to lay their eggs (*t*-test, *t*_(4)_ = -1.3312, *P* = 0.2539). However, females laid significantly fewer eggs in beakers with p-cresol at 10^-4^ ppm (*t*-test, *t*_(4)_ = 11.435, *P* = 0.0003), 10^1^ ppm (*t*-test, *t*_(4)_ = 4.391, *P* = 0.0118) and 10^-2^ ppm (*t*-test, *t*_(6)_ = 10.344, *P* < 0.0001). Concerning the three-way ANOVA model, there was no significant effect of the factors “Date” (*F*_(9,__11)_ = 1.312, *P* = 0.33021) or “Egg number” (*F*_(1,11)_ = 0.800, *P* = 0.39012), meaning that the blood meal and number of eggs laid had no effect on female choices. The “Choice” factor was significant (*F*_(4,11)_ = 9.609, *P* = 0.00136).

## Discussion

Manual counting mosquito eggs is a challenge requiring substantial effort and time. Automatic and semi-automatic tools for egg counting bring advantages such as reducing working time, greater consistency, and reduction of experimenter bias. Our results show that using ICount software to process sandpaper strips and count thousands of eggs required less than half an hour to process all the sandpaper strips (*n* = 48). This is a substantial improvement over the ≥ 5 min per oviposition sandpaper required for manual counting (see Table 1). The software also helps the experimenter by saving the threshold adjustments and egg detection parameters for different images, contrary to the automatic ImageJ method used by Afify & Galizia [22], where each image needs threshold adjustments. Reliability of the two automatic methods was comparable with 2% (ICount) and 3% (ImageJ) errors with pictures containing less than 100 eggs. For pictures containing more than 200 eggs, the error rate was of 12% for ICount and 14 for ImageJ.

Our results showed that ICount software offers an excellent option as a useful tool for egg quantification across a broad range of egg numbers. The accuracy can be brought to 100% if the procedure is semi-automatized. Semi-automatic systems are a combination of automatic processing with manual tunings or corrections [17]. In ICount, the user can change the tuning of parameters used by the automatic part of the system, such as the threshold used in the binary image and the maximum/minimum object size. This method allows the user to significantly improve the accuracy of the estimates. This approach should be chosen when precise egg numbers are more important than the data analysis speed. After each count, ICount software tags each egg with a green square when the certainty of counting is above the set threshold. However, when the algorithm detects an object under this threshold, the object is tagged with a red square (see Fig. 2b). The user can then check if the object is an artefact (overestimation) or multiple objects superposed (underestimation). The egg estimation by the software using this method can reduce errors to zero.

Other automatic counting methods have been suggested in the past. However, most of them require a scanner for egg sampling, which is not practical for field analysis [12, 14, 16, 22]. Moreover, images taken with scanners need complex image processing using ImageJ before automatic counting. Other studies suggest methods from camera images but automated counting is not straightforward and needs image segmentation before the number of eggs can be estimated [12, 14]. The technique described here requires only a camera, no complex image processing and has a user friendly interface. ICount is also a free software which can be downloaded [18]. Similarly, “Egg-counter”, designed to automatically count eggs from *Anopheles* mosquitoes, was released in 2014 [12]. However, our attempts to process our “micro” or “macro” images with this free software, detected no eggs. This may be because threshold and object detection settings in the software cannot be modified.

The limitations of this technique lie in the visual processing of the image rather than development of the algorithm. For ICount, any flexible support where females can lay their eggs can be processed, as long as the contrast between background (light colour) and the eggs (black) is sharp enough. In this case, pictures of the eggs are the only requirement and do not require complex processing. In order to test the limits of ICount, different supports with various colours have been given to females to lay their eggs. It seems that colour contrast is not the limitation of the software since the threshold can be adjusted manually, but rather the type of support which is used*.* Indeed, *Ae. aegypti* females are more likely to lay eggs into “clusters” (meaning more than 3 eggs touching each other) on blotting paper than sandpaper, filter and tissue papers (see Additional file 2: Table S1). This could explain why blotting paper supports have higher percentage errors (>35% for “micro” images) when processed with ICount, compared to the other supports used (< 20% for “micro” images). An additional limitation appears with eggs laid on sandpaper strips when total egg number is above 150 and 1000 eggs per “micro” and “macro” images, respectively (see Fig. 3). The increase of counting errors is probably due a high number of eggs laid on the restricted surface. When egg density increases the probability of eggs touching and laid next to each other increases. Egg clusters can be avoided by providing more surface to females to lay their eggs or replacing the egg support more often.

Given a minimal quality threshold for definition, ICount is not limited by the image format; it works effectively with images of the overall sandpaper or filter paper strip taken with a regular camera as well as very high quality pictures taken with a microscope in the laboratory. ICount constitutes a fast and practical tool to sample *Ae. aegypti* eggs in the laboratory and/or in the field.

In this study, the software was also used to quantify *Ae. aegypti* female oviposition choices. P-cresol is known to influence the egg laying choices of female mosquitoes [3]. Here, we have shown that, under laboratory conditions, females were repulsed by containers with p-cresol at 10^-2^ ppm and 10^-4^ ppm. This confirms the literature on the repulsive effect of p-cresol at low concentrations [22]. The effect disappears at 10^-1^ ppm where no preference was observed between p-cresol and water, as shown previously [22]. A significant repulsive effect is also observed at 10^1^ ppm, meaning that the range of neutral tolerance of *Ae. aegypti* for p-cresol is between 10^-2^ ppm and 10^1^ ppm. This data not only reinforces knowledge of *Ae. aegypti* preferences, but also shows that ICount can be easily used to acquire data of this type of behaviour experiment. Indeed, the semi-automatic method allowed both a fast and precise estimation of the number of eggs laid in each condition.

ICount can be also used to count eggs from other *Aedes* species, which increases its field of application. The software has been tested with “macro” images of *Aedes albopictus* eggs (*n* = 5 sand paper strips, see Additional file 3: Table S1 and Figure S1). We found an average error of 7.4% (± 4%), indicating that the method could be extended to other *Aedes* species. Eggs belonging to the two different *Aedes* species could not be distinguished with ICount; however, this could be a good option to develop with higher definition images for species recognition via egg characteristics. Assays with eggs from *Anopheles* species could not be performed as our insectary is not currently suitable for rearing *Anopheles* colonies. Trials have been done with *Culex* mosquitoes. Despite their different strategy to lay their eggs, which are deposited on the surface of the water into rafts. The ICount algorithm cannot distinguish individual eggs clustered into rafts but it can automatically count the number of rafts laid on the surface of the water (see Additional file 3: Figures S2 and S3 for illustration). Although single *Culex* eggs cannot be distinguished by ICount, the software could still be used for vector control strategies to estimate the attractiveness of specific oviposition sites by counting the number of rafts laid by *Culex* vectors.

**Fig. 5:**
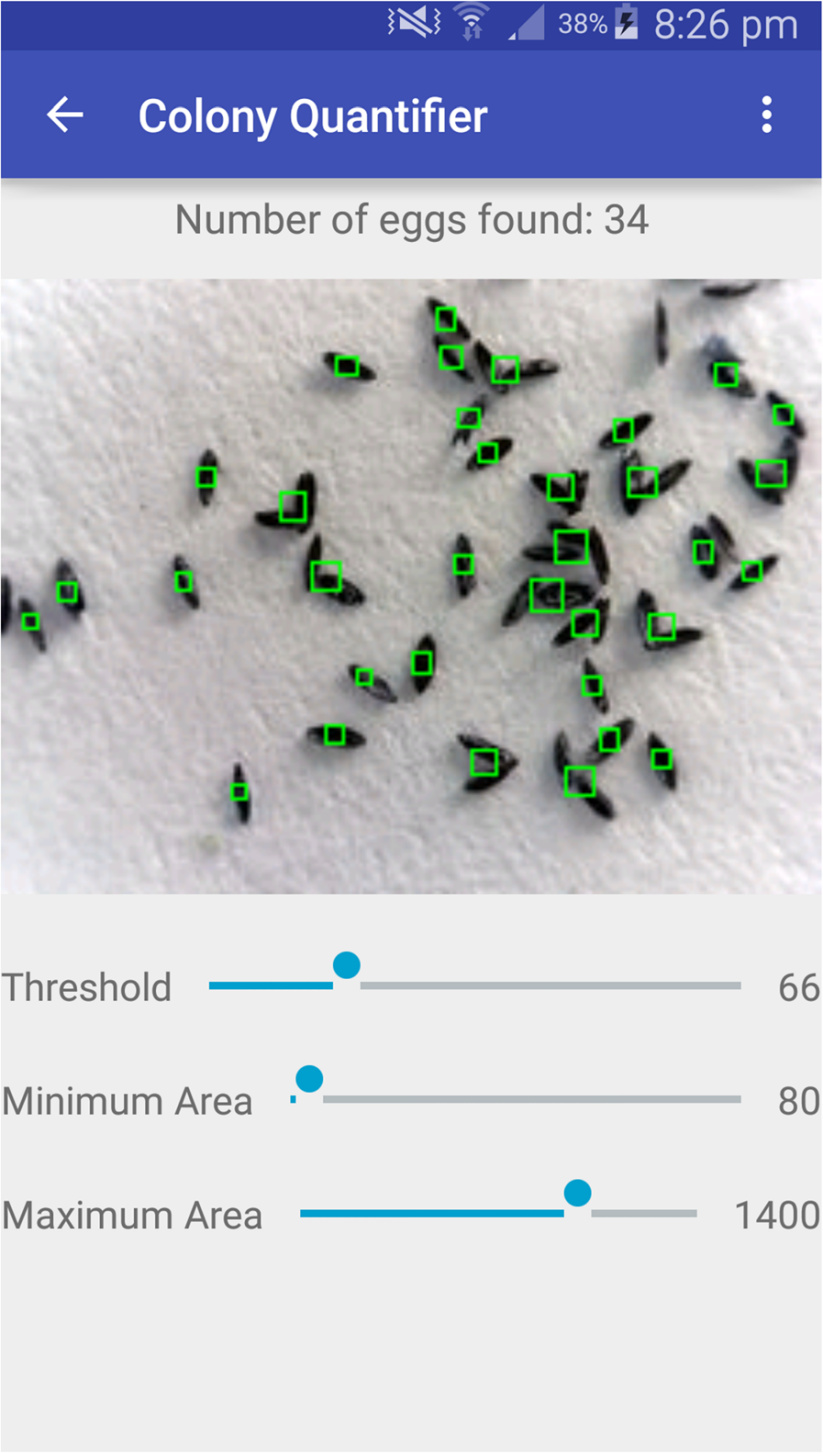
Screenshot of ICount mobile application interface. Pictures from eggs can be taken with a mobile integrated camera and directly counted with the application

The next step for the development of ICount, with minimalist syntax and features, will be to develop a mobile application in order to use the automatized method in the field (see Fig. 5). This could help global surveys by acquiring and recording the data directly on site with time (date) and space (GPS) parameters and quickly respond to treat sites which show high abundance of mosquito vectors. Use of very light and cheap material (a mobile phone with camera) would make the use of the method accessible at any site by skilled or semi-skilled operators. Moreover, transferring paper-based surveillance data is a slow process, while an electronic database can prevent delays for completion of data analyses [26]. During outbreaks, surveillance data and rapid data transfer to a central database are important to increase the statistical power of data analysis and provide a rapid public health response. Mobile data sampling, using such software, represents a powerful tool to increase vector-borne disease surveillance and could help to evaluate the impact of vector control interventions.

## Conclusion

Free and user friendly software, ICount, has been validated for automatic and semi-automatic counting of *Ae. aegypti* eggs. Also validated with p-cresol assays in our laboratory, this new tool allows a rapid and precise data sampling and analysis. ICount is the first step in developing a mobile application for automatic egg counting. Development of such new tools are important for vector surveillance in the field, especially in the context of recent vector-borne disease outbreaks.

## Additional files

Additional file 1: ICount limitation, examples of low and high egg densities automatically counted with the software. **Figure S1.** Illustration with “Micro” pictures. **Figure S2.** Illustration with “Macro” pictures. (DOCX 5469 kb)
Additional file 2: ICount efficiency testing with different types of support for mosquito females to lay their eggs. **Table S1.** Efficiency calculated in percentage for each type of support with “Micro” and “Macro” pictures. **Figure S1.** Illustration of automatic egg counting with different types of support with Icount. (DOCX 9024 kb)
Additional file 3: Icount assessment with eggs laid from different vector species. **Table S1.** Icount efficiency in percentage of error for counting *Aedes albopictus* eggs laid on sand papers strips (Manual versus automatic counting). **Figure S1.** Illustration of *Aedes albopictus* eggs processed with Icount using a “Macro” picture. **Figure S2.** Illustrations of *Culex quinquefasciatus* egg rafts laid on water in a small plastic pot with ICount. **Figure S3.** Illustrations of *Culex annulirostris* egg rafts laid on water in a glass Petri dish with ICount. (DOCX 3159 kb)

## Abbreviation

P-cresol: 4-methylphenol
