# Supplementary material for: Assessment of ICount software, a precise and fast egg counting tool for the mosquito vector Aedes aegypti
Source: Parasit Vectors. 2016 Nov 18;9:590. doi: 10.1186/s13071-016-1870-1 (PMC5116143; doi:10.1186/s13071-016-1870-1)
Supplement: Additional file 1: — ICount limitation, examples of low and high egg densities automatically counted with the software. Figure S1. Illustration with “Micro” pictures. Figure S2. Illustration with “Macro” pictures. (DOCX 5469 kb) [file 13071_2016_1870_MOESM1_ESM.docx]

**Additional file 1**

**ICount limitation: high egg density and overlapping**

**Figure S1: “Micro” images at different egg densities illustrating the overlapping limitation.**

“Micro” image at low density (egg number < 50 per image)

**
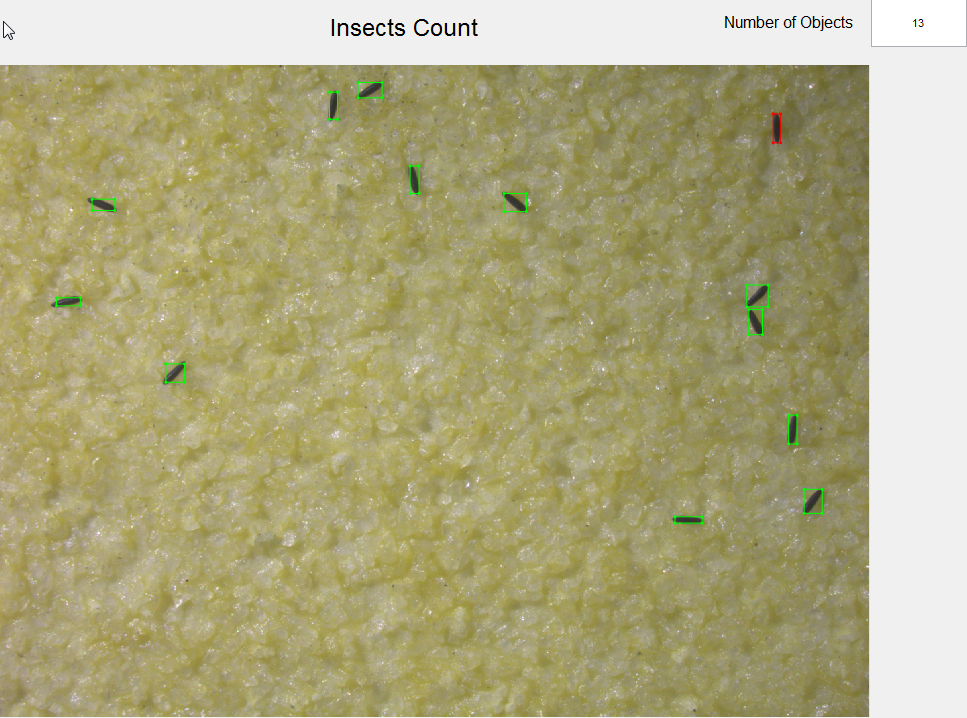
**

Manual counting: Number of eggs = 13

“Micro” image at low density but overlapping

**
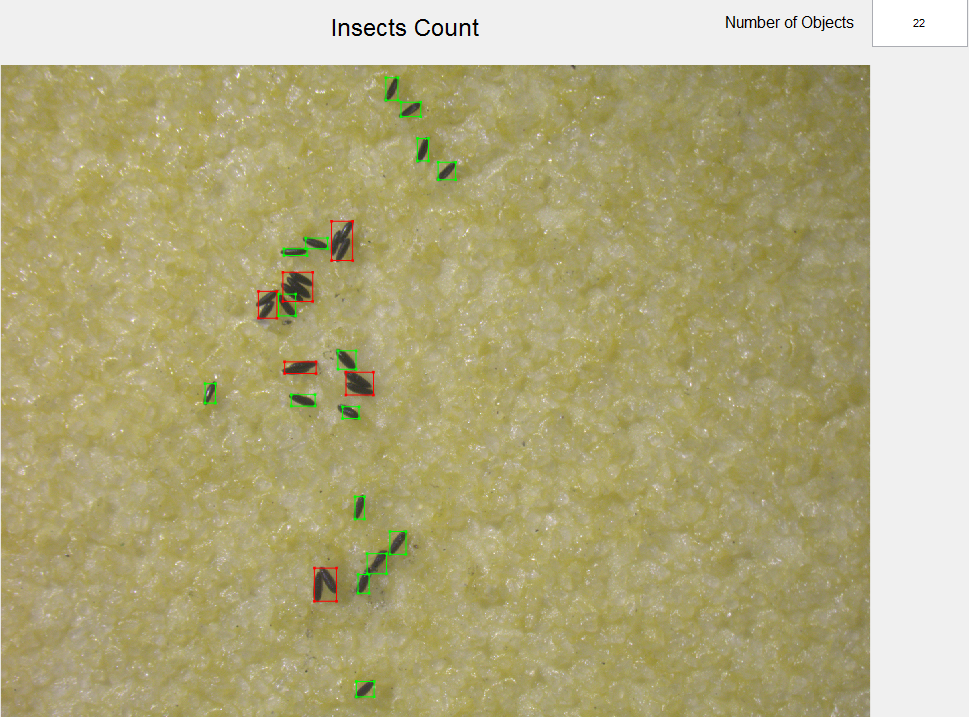
**

Manual counting: Number of eggs = 29

“Micro” image at high density (egg number > 200 per image)

**
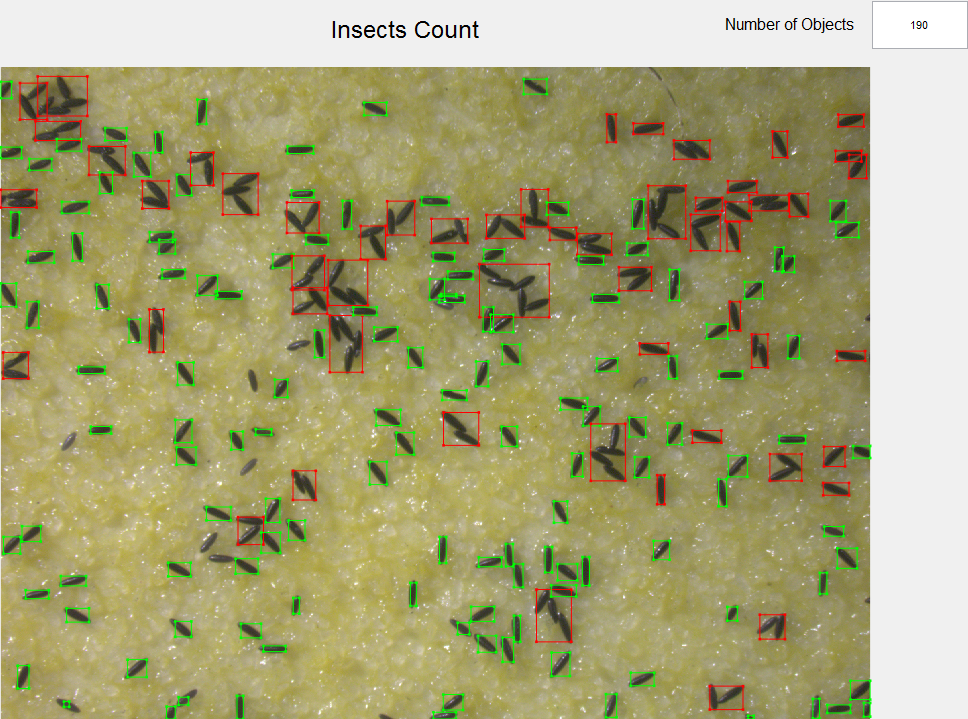
**

Manual counting: Number of eggs = 228

**Figure S2: “Macro” images at different egg densities illustrating the overlapping limitation.**

“Macro” image with low density (egg number <200 per image)

**
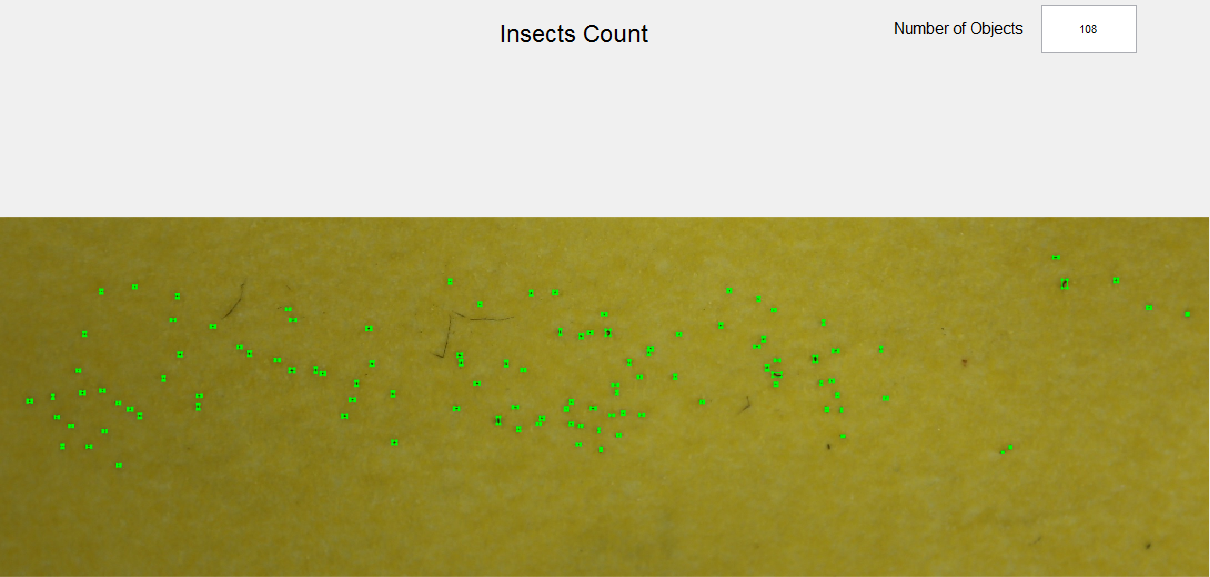
**

Manual counting: Number of eggs = 110

“Macro” image with high density (egg number >1000 per image)

**
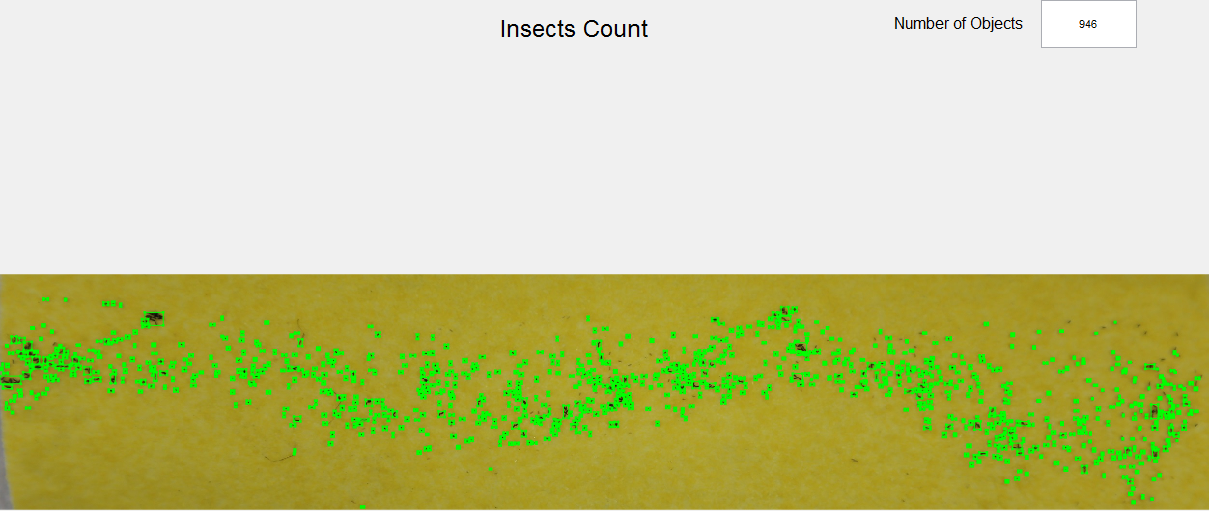
**

Manual counting: Number of eggs = 1092
