# Supplementary material for: Assessment of ICount software, a precise and fast egg counting tool for the mosquito vector Aedes aegypti
Source: Parasit Vectors. 2016 Nov 18;9:590. doi: 10.1186/s13071-016-1870-1 (PMC5116143; doi:10.1186/s13071-016-1870-1)

**Additional file 2**

**Different supports for ICount efficiency testing**

**Table S1**

Summary table of ICount efficiency (“micro” and “macro” pictures) with different supports for egg laying.

| **Supports** | **Error micro images (%)** | **Error macro images (%)** |
| --- | --- | --- |
| Coffee filter | 19.65 | 29.48 |
| Blue blotting paper | 52.62 | 32.74 |
| Red blotting paper | 39.53 | 26.18 |
| Green blotting paper | 37.30 | 44.90 |
| White tissue paper | 18.14 | 12.51 |

**Figure S1: Different supports (colour and texture) tested with Icount for egg counting**

Support 1: coffee filter (brown)

“Micro” picture


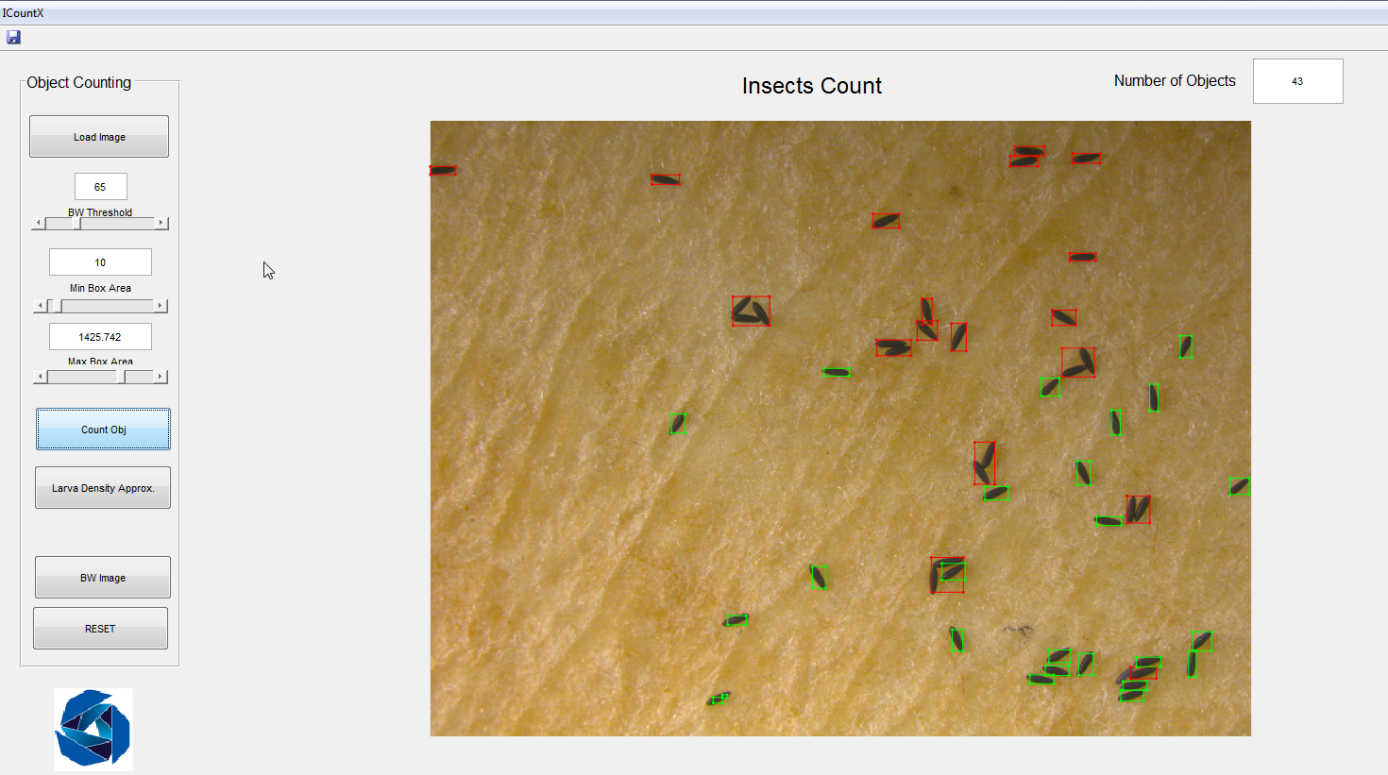


“Macro” picture


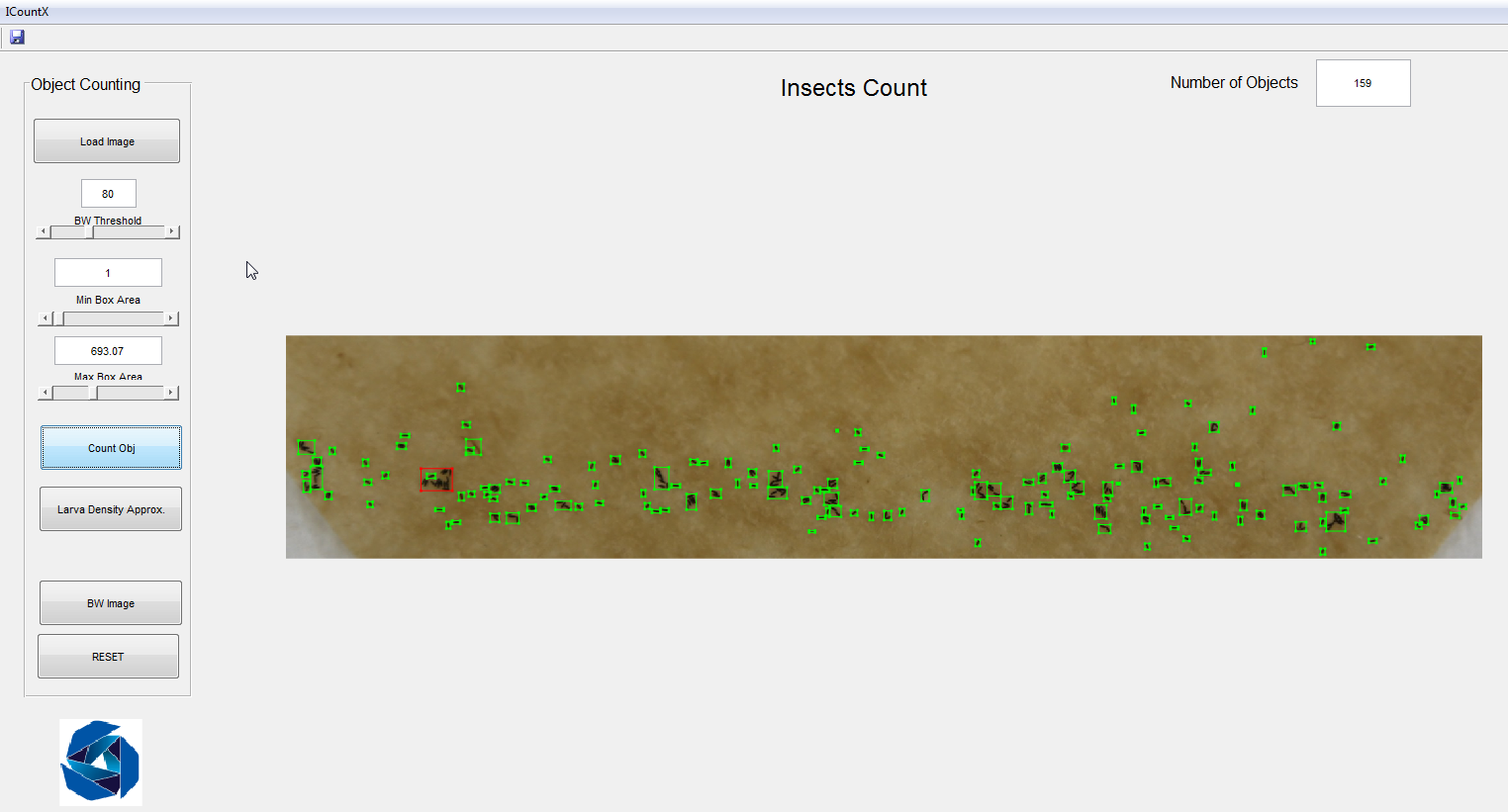


Support 2: blotting paper (blue)

“Micro” picture


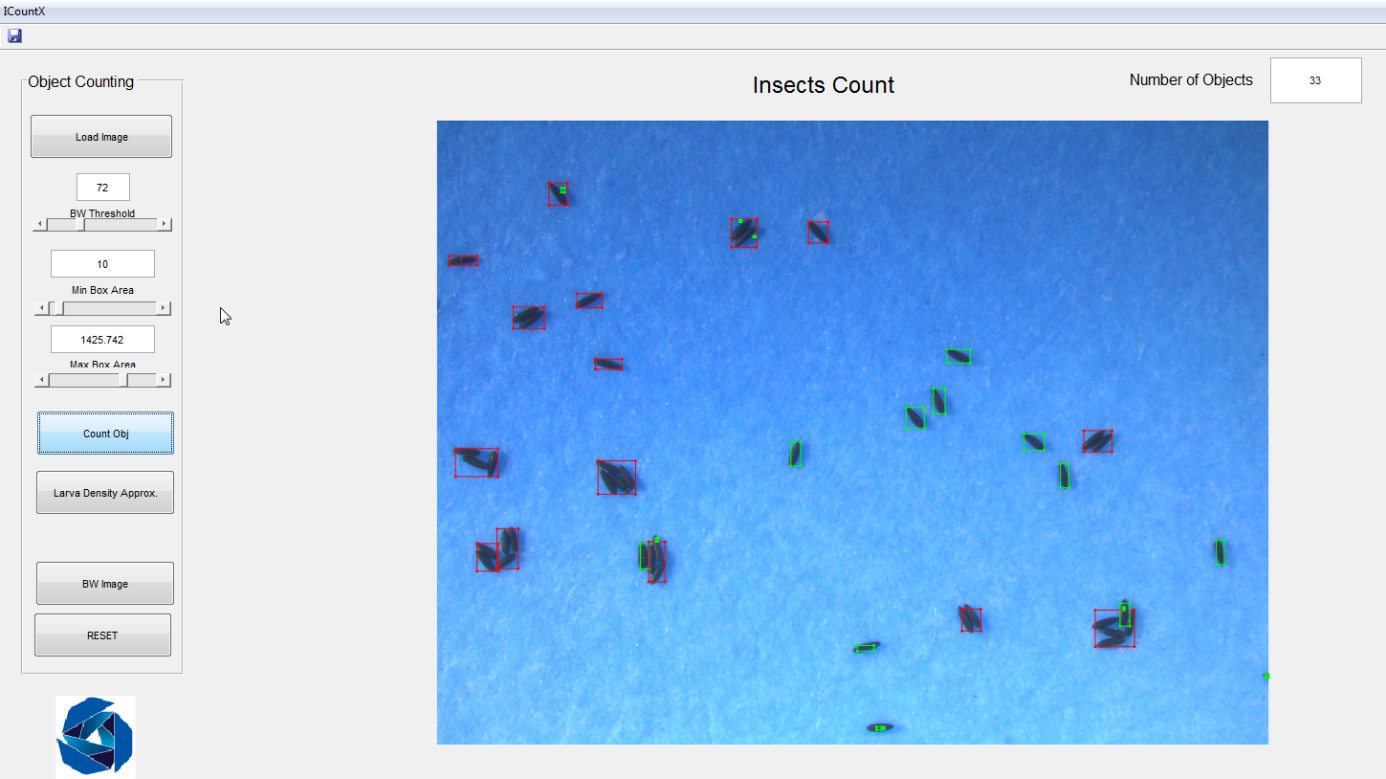


“Macro” picture


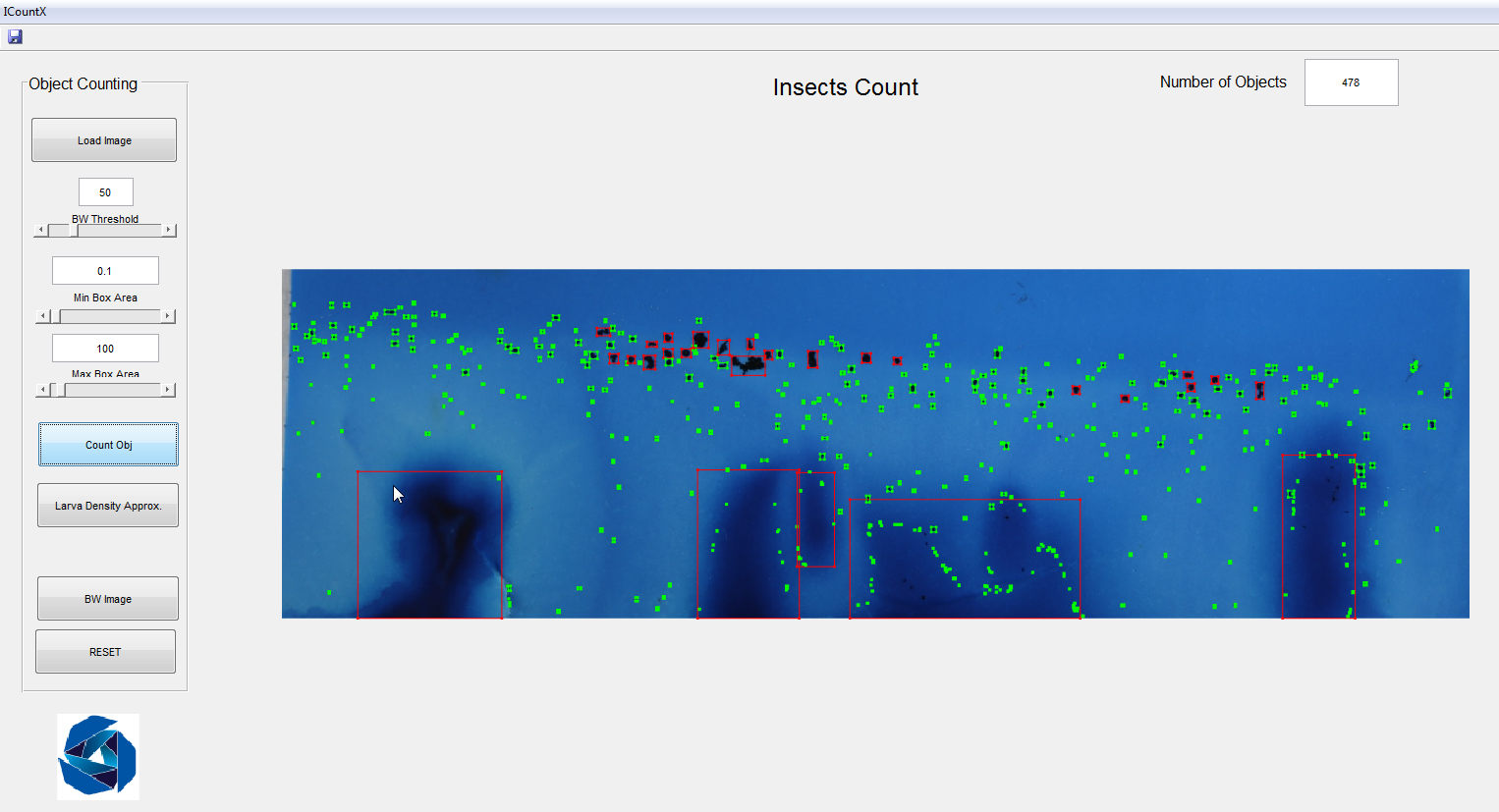


Support 3: blotting paper (red)

“Micro” picture


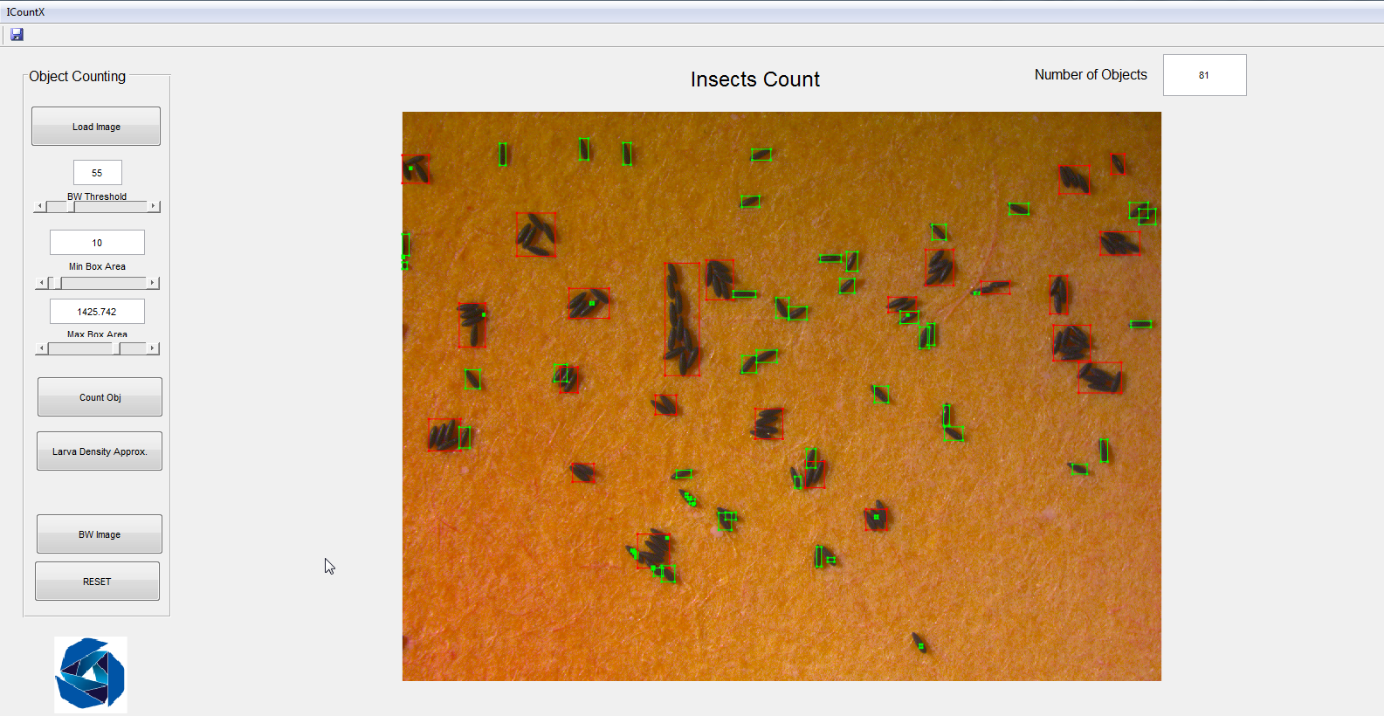


“Macro” picture


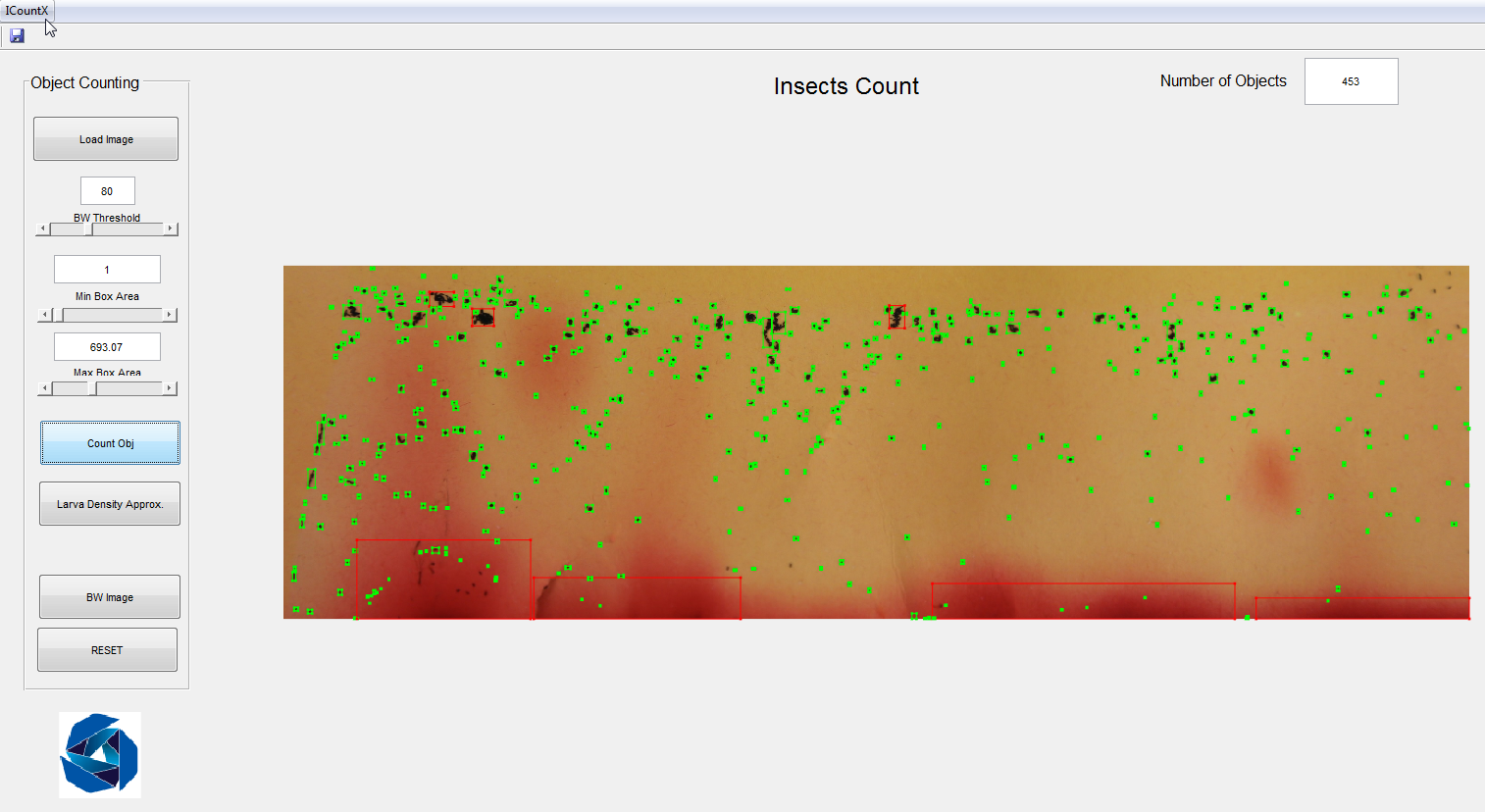


Support 4: blotting paper (green)

“Micro” picture


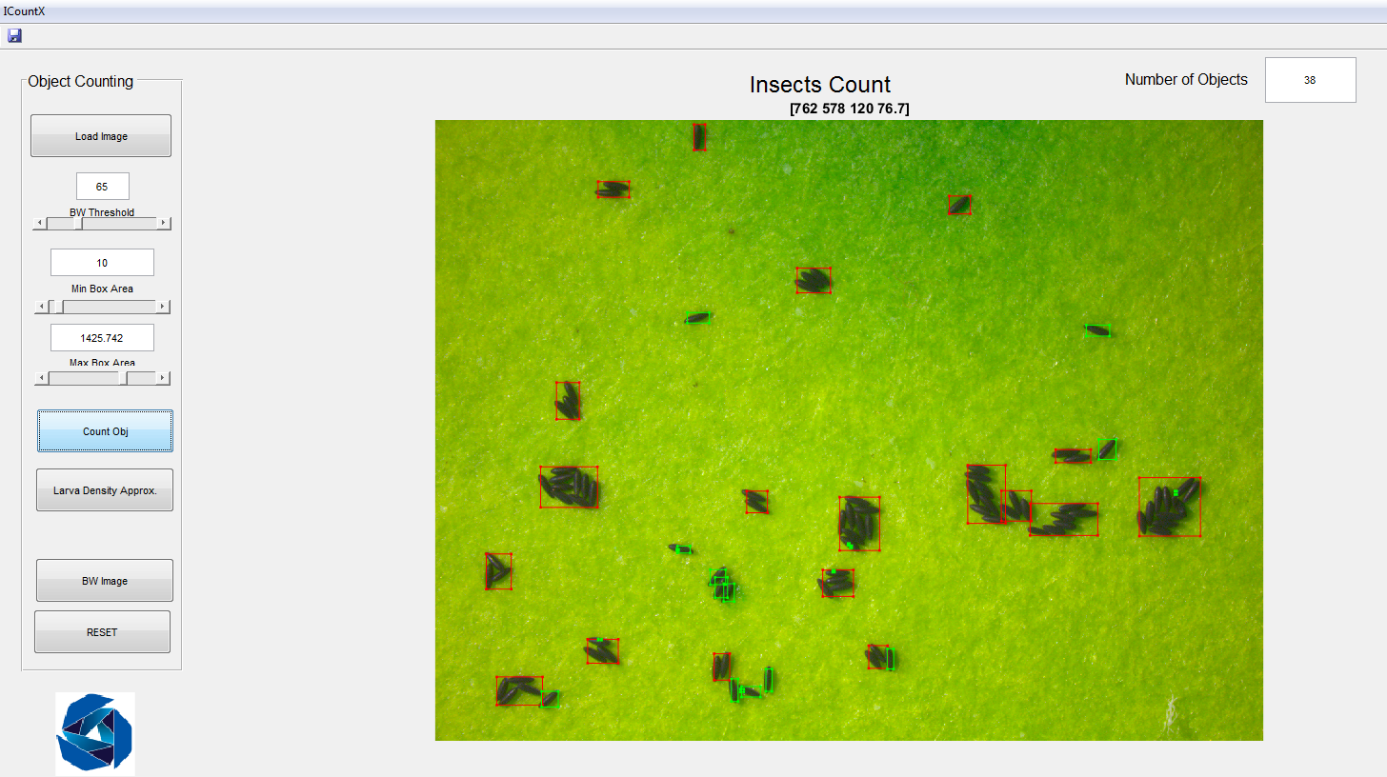


“Macro” picture


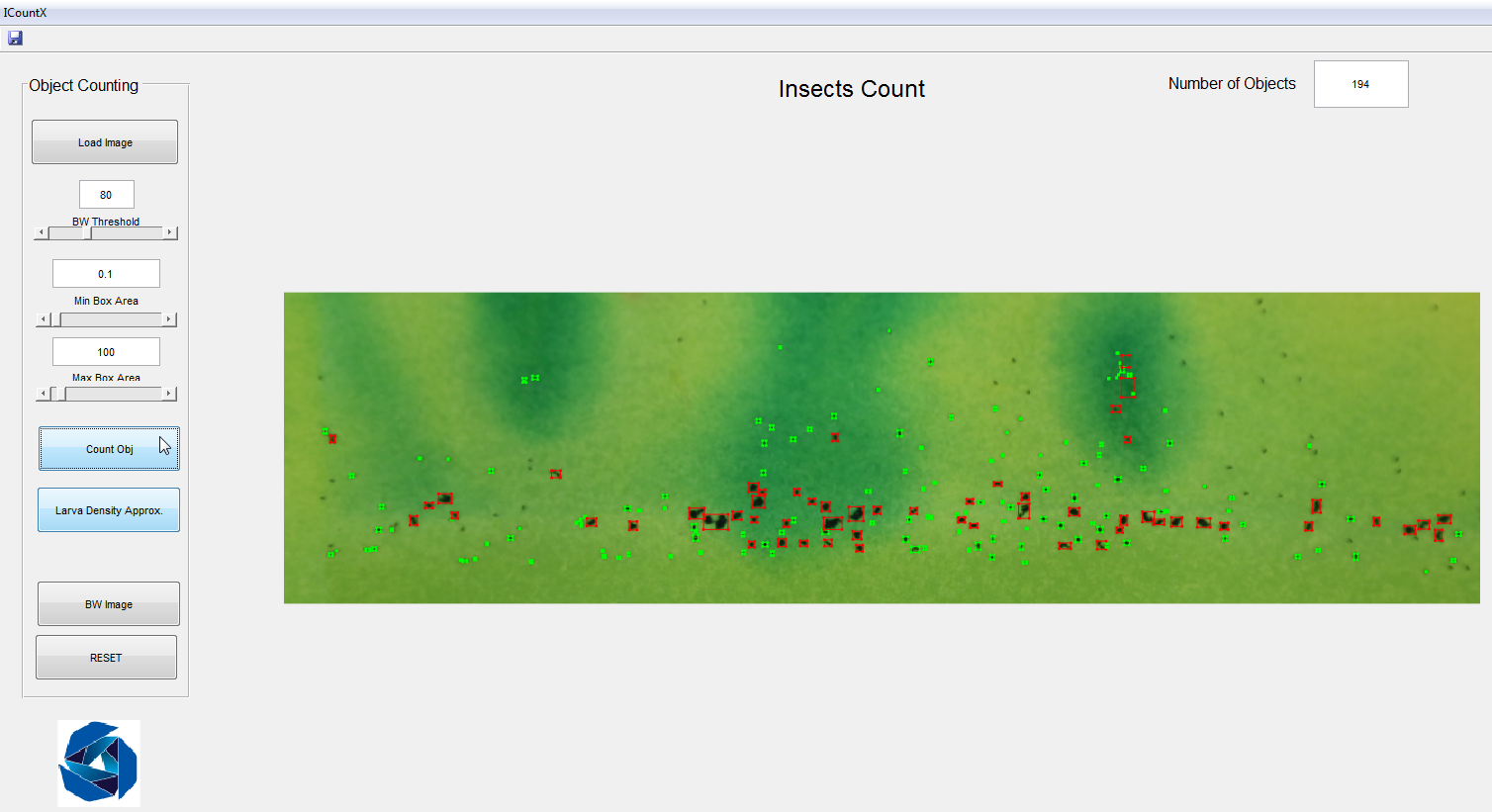


Support 5: tissue paper (white)

“Micro” picture


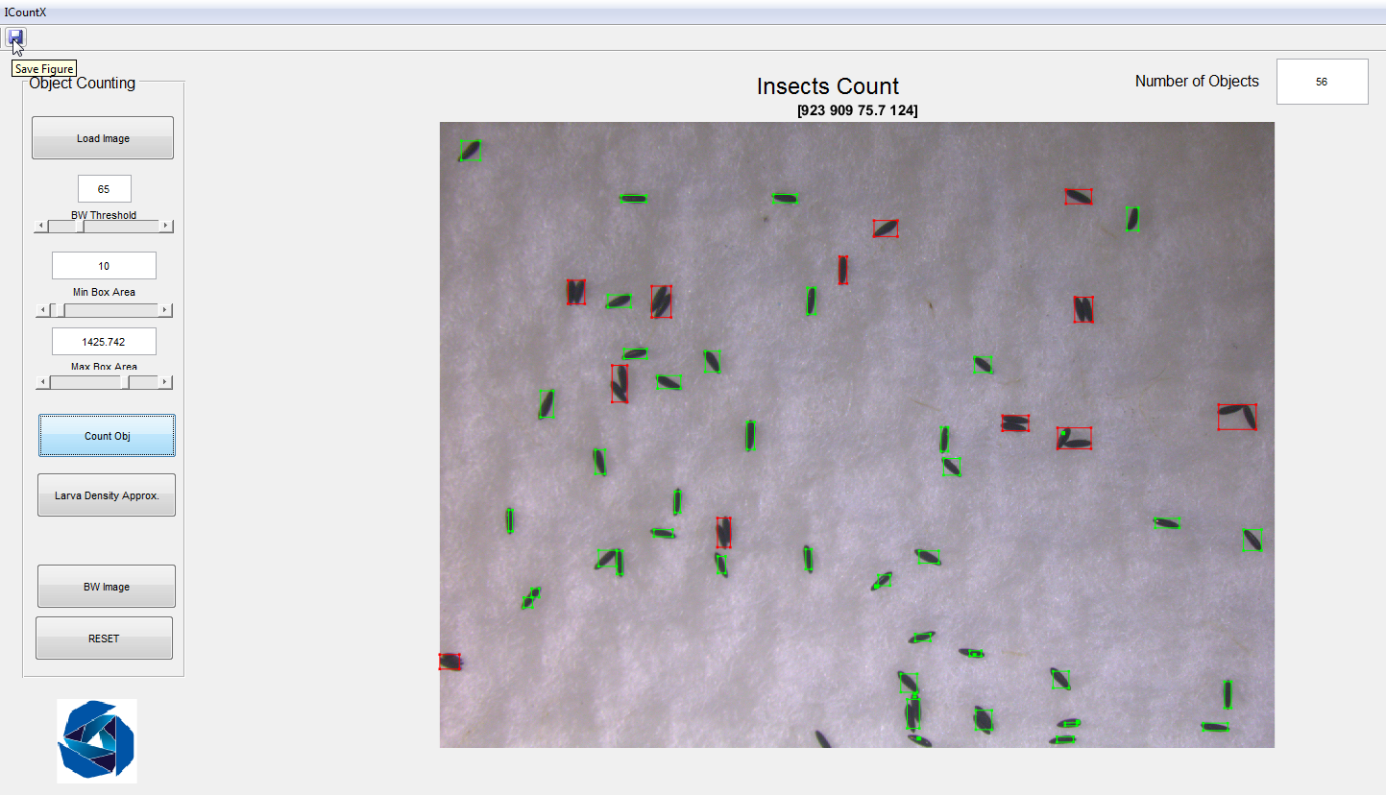


“Macro” picture


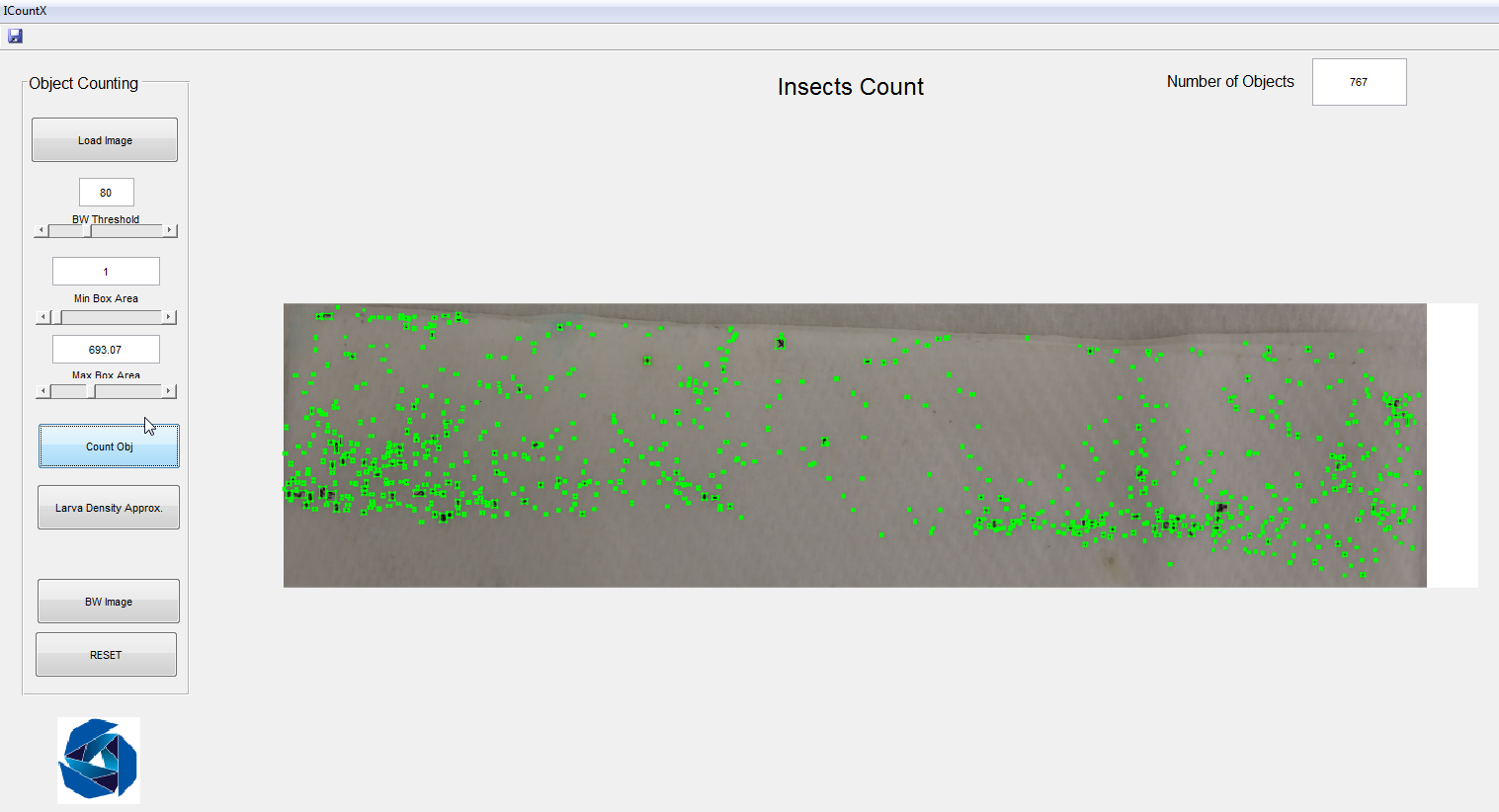

Supplement: Additional file 2: — ICount efficiency testing with different types of support for mosquito females to lay their eggs. Table S1. Efficiency calculated in percentage for each type of support with “Micro” and “Macro” pictures. Figure S1. Illustration of automatic egg counting with different types of support with Icount. (DOCX 9024 kb) [file 13071_2016_1870_MOESM2_ESM.docx]
