# Supplementary material for: Assessment of ICount software, a precise and fast egg counting tool for the mosquito vector Aedes aegypti
Source: Parasit Vectors. 2016 Nov 18;9:590. doi: 10.1186/s13071-016-1870-1 (PMC5116143; doi:10.1186/s13071-016-1870-1)

**Additional file 3**

**ICount assessment with different vector species**

**Table S1**

Summary table of ICount efficiency with *Aedes albopictus* eggs laid on sand papers.

| **Images #** | **Manual** | **Auto** | **Error** | **% error** |
| --- | --- | --- | --- | --- |
| 1 | 383 | 371 | 25 | 3.13 |
| 2 | 80 | 89 | 9 | 11.25 |
| 3 | 120 | 127 | 7 | 5.83 |
| 4 | 165 | 187 | 22 | 13.33 |
| 5 | 168 | 174 | 6 | 3.57 |
| **Average** |  |  |  | 7.42 (±4.04) |

**Figure S1**

“Macro” image of *Ae. Albopictus* eggs, processed with Icount.


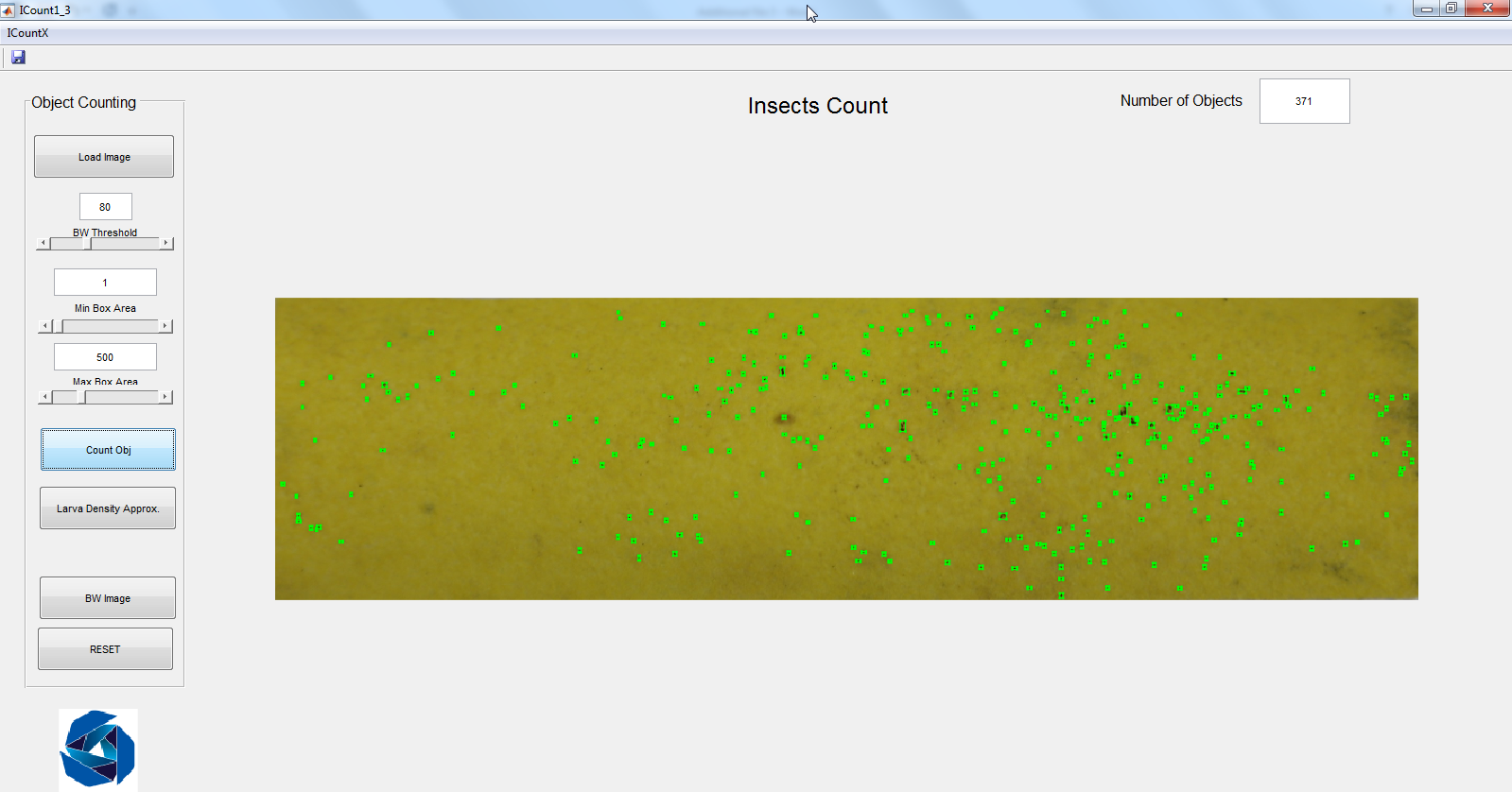


**Figure S2**

Rafts from *Culex quinquefasciatus* processed into ICount for automatic raft counting.


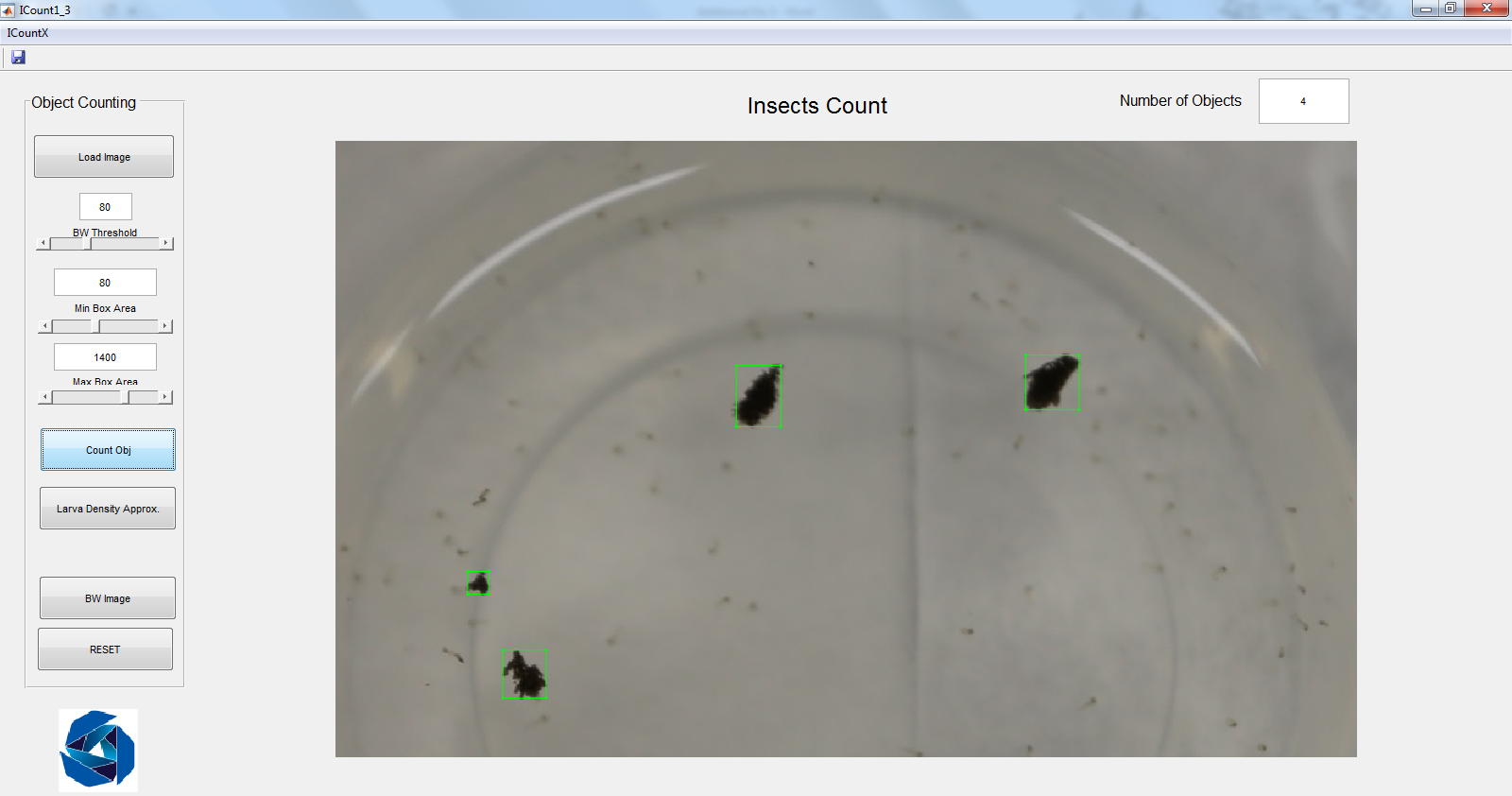


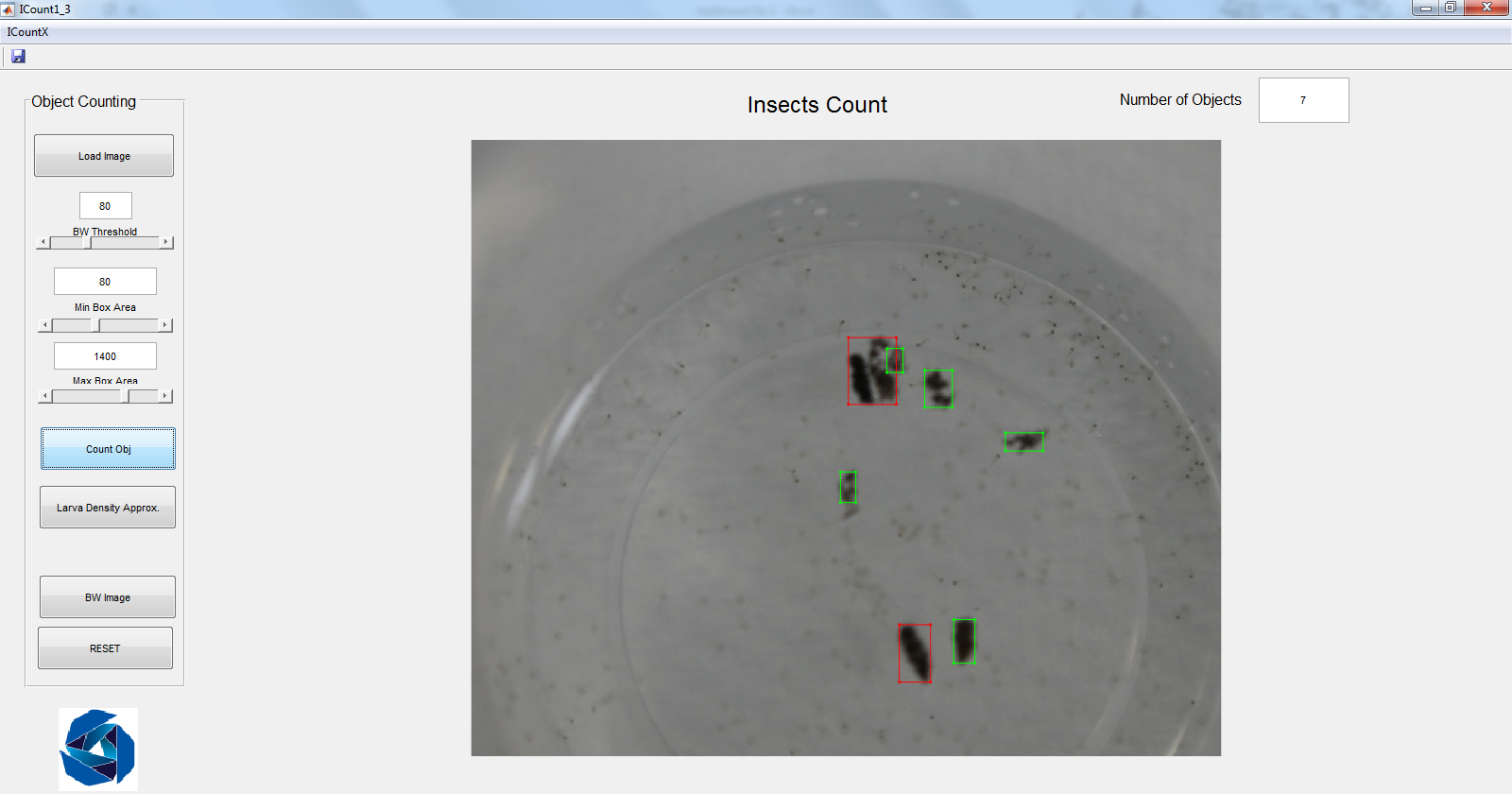


**Figure S3**

Rafts from *Culex annulirostris* processed into ICount for automatic raft counting.


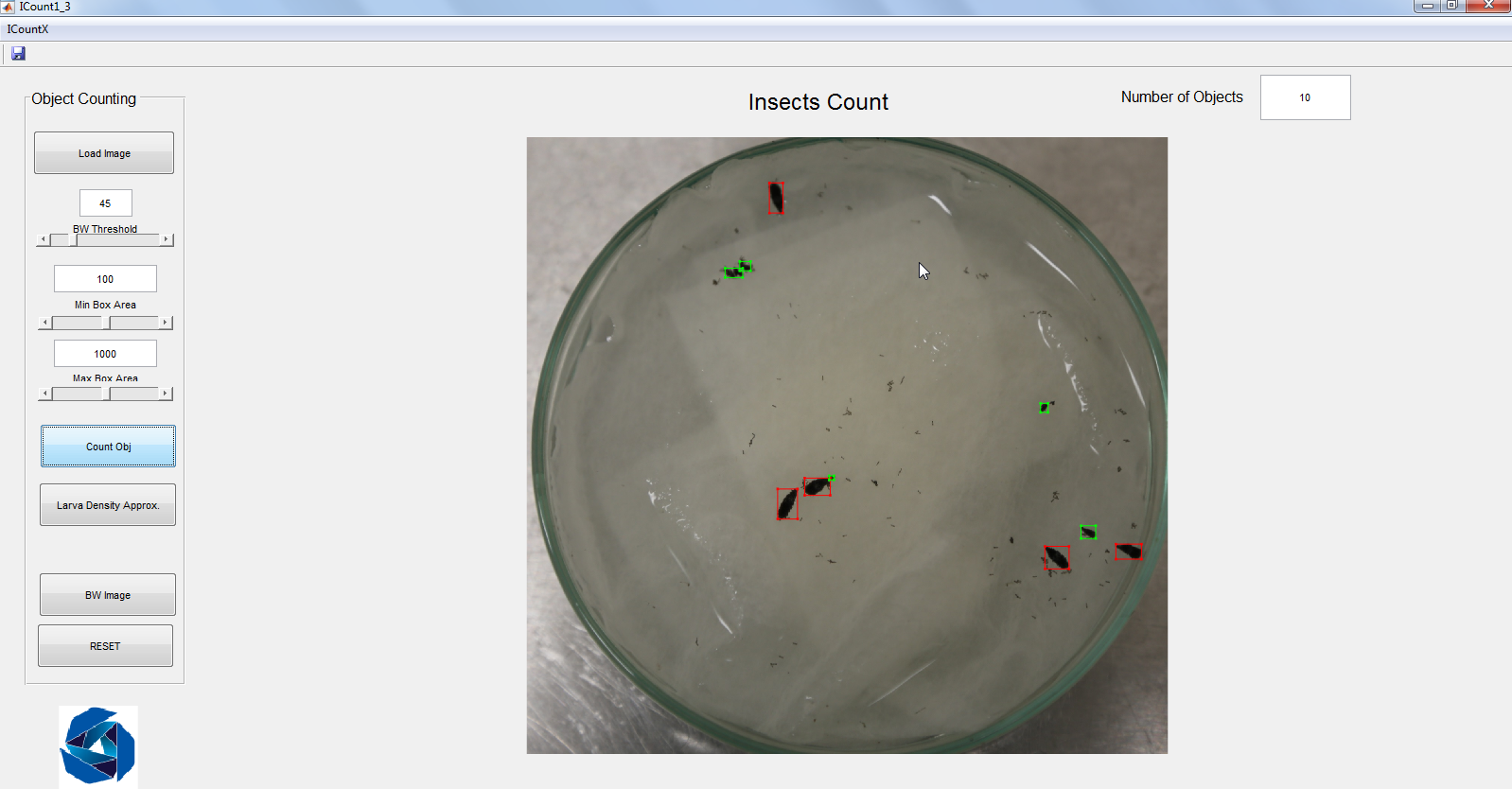


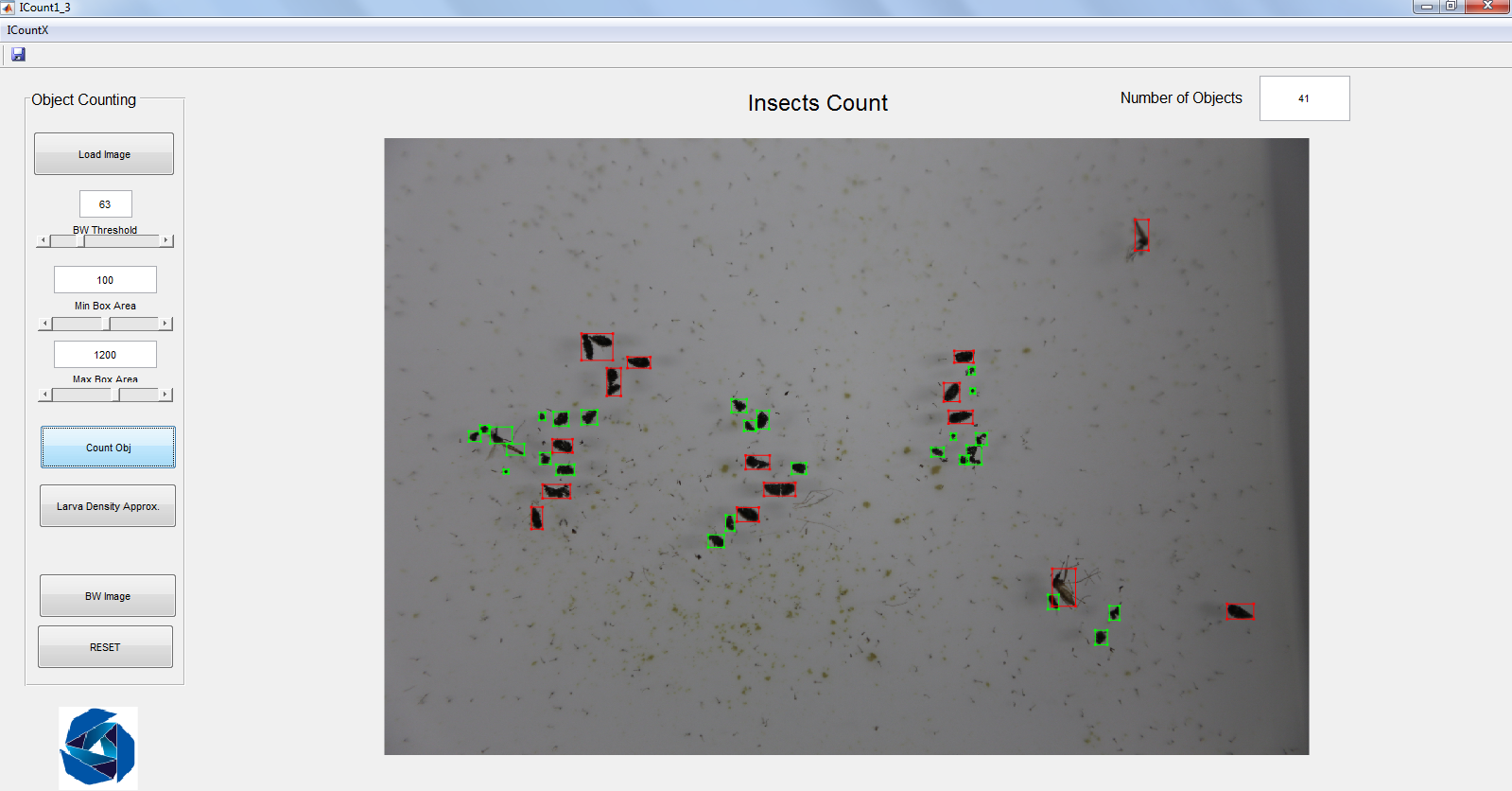

Supplement: Additional file 3: — Icount assessment with eggs laid from different vector species. Table S1. Icount efficiency in percentage of error for counting Aedes albopictus eggs laid on sand papers strips (Manual versus automatic counting). Figure S1. Illustration of Aedes albopictus eggs processed with Icount using a “Macro” picture. Figure S2. Illustrations of Culex quinquefasciatus egg rafts laid on water in a small plastic pot with ICount. Figure S3. Illustrations of Culex annulirostris egg rafts laid on water in a glass Petri dish with ICount. (DOCX 3159 kb) [file 13071_2016_1870_MOESM3_ESM.docx]
